# VE-Cadherin modulates β-catenin/TCF-4 to enhance Vasculogenic Mimicry

**DOI:** 10.1038/s41419-023-05666-7

**Published:** 2023-02-17

**Authors:** Daniel Delgado-Bellido, Esteban Zamudio-Martínez, Mónica Fernández-Cortés, Ana Belén Herrera-Campos, Joaquin Olmedo-Pelayo, Carmen Jordán Perez, José Expósito, Enrique de Álava, Ana Teresa Amaral, Francisco O’ Valle, Angel Garcia Diaz, F. J. Oliver

**Affiliations:** 1 Instituto de Parasitología y Biomedicina López Neyra, CSIC, Granada, Spain; 2 Instituto de Salud Carlos III, CIBERONC, Madrid, Spain; 3 Instituto de Biomedicina de Sevilla, Hospital Virgen del Rocío, Seville, Spain; 4 Complejo Hospitalario de Granada, PTS de Granada, Granada, Spain

**Keywords:** Adherens junctions, Oncogenesis

## Abstract

Vasculogenic Mimicry (VM) refers to the capacity to form a blood network from aggressive cancer cells in an independent way of endothelial cells, to provide nutrients and oxygen leading to enhanced microenvironment complexity and treatment failure. In a previous study, we demonstrated that VE-Cadherin and its phosphorylation at Y658 modulated kaiso-dependent gene expression (CCND1 and Wnt 11) through a pathway involving Focal Adhesion kinase (FAK). In the present research, using a proteomic approach, we have found that β-catenin/TCF-4 is associated with nuclear VE-cadherin and enhances the capacity of malignant melanoma cells to undergo VM in cooperation with VE-Cadherin; in addition, preventing the phosphorylation of Y658 of VE-cadherin upon FAK disabling resulted in VE-Cadherin/β-catenin complex dissociation, increased β-catenin degradation while reducing TCF-4-dependent genes transcription (C-Myc and Twist-1). Uveal melanoma cells knockout for VE-Cadherin loses β-catenin expression while the rescue of VE-Cadherin (but not of the phosphorylation defective VE-Cadherin Y658F mutant) permits stabilization of β-catenin and tumor growth reduction in vivo experiments. In vivo, the concomitant treatment with the FAK inhibitor PF-271 and the anti-angiogenic agent bevacizumab leads to a strong reduction in tumor growth concerning the single treatment. In conclusion, the anomalous expression of VE-Cadherin in metastatic melanoma cells (from both uveal and cutaneous origins), together with its permanent phosphorylation at Y658, favors the induction of the aggressive VM phenotype through the cooperation of β-catenin with VE-Cadherin and by enhancing TCF-4 genes-dependent transcription.

## Introduction

Vasculogenic Mimicry or Vascular Mimicry (VM) characterizes the capacity of establishing perfusion gateway in a variety of tumors by highly invasive, genetically deregulated tumors cells: vasculogenic because they diffuse plasma and red blood cells that flow around tumor cells and mimicry by considering that vessels are not authentic blood vessels and exclusively mimic the vascular function in an angiogenesis dependent manner but not excluding both pathways. Although VM formation is an indicator of highly invasive can cell phenotype, the mechanisms by which this particular configuration may participate to negatively affect the tumor outcome are not well understood. It has been suggested that VM formation may facilitate possible tumor perfusion and the physical connection between VM and blood vessels may also help the hematogenous propagation of tumor cells. There is a robust correlation between the histological presence of VM patterns in primary uveal and cutaneous melanoma and the resulting death by metastasis progression [1–3] constant with the in vitro inspection that these patterns are induced solely by highly invasive tumor cells [4]. Endothelial Cells (ECs) previously exclusively express different members of the cadherin superfamily, specifically, vascular endothelial VE-cadherin, which is the principal adhesion receptor of endothelial adherent junctions. Non-endothelial or extra-vascular expression of VE-cadherin has been observed in specific cancer types associated with VM [5]. VM was first described in uveal melanoma; more recently, and following the rare aspect of this type of tumor, several seminal articles described by a platform of synthetic lethal gene interaction networks, that approximately 80–90% of uveal melanoma have a GNAQ-GNAQ11 mutation [6] leading to constitutively active Gαq proteins which in turn render them as driver oncogenes and this aberrant mutation in this rare cancer produce a unbalance mechanism of downstream kinases. Indeed, the alteration in the activity of the kinase FAK (Y397) is the most important change derived from the aberrant expression of GNAQ protein in the membrane of the cell, affecting localization and phosphorylation of YAP (pY357) and enhancing the activity of the transcription factor YAP in the nucleus [3, 7].

The Wnt/*β*-catenin signaling pathway is crucial to confer homeostasis of the cells and embryonic progress; it was significantly proven to be associated with tumor cell proliferation, apoptosis, invasion, self-renewal, and chemotherapy resistance in distinct tumor models, particularly in colon cancer [8, 9]. Wnt signaling is crucial in cell growth control and plays a role in many biological processes extending from progress and evolution to adult homeostasis. Wnt signaling covers two different ramifications: canonical (β-catenin-dependent activity) and non-canonical (β-catenin-independent activity) Wnt pathways. β-catenin supplies the backbone of the adherents junction in the endothelium, including twelve armadillo repeats flanked by similar domains in the N- and C-terminus, separately [10]. It acts as a critical nuclear effector of the canonical Wnt pathway. In the absence of a Wnt ligand, cytoplasmic β-catenin is controlled by the Adenomatous polyposis coli (APC), Axis Inhibition protein (AXIN), Glycogen Synthase Kinase 3 (GSK3) and Casein Kinase 1 (CK1) for β-catenin elimination and the nuclear re-localization with transcription consequences [11, 12]. Once stabilized, cytoplasmic β-catenin translocates to the nucleus where it binds to transcription factors, such as the TCF/LEF family [13]. In the current study, we elucidated a novel mechanism involved in VM development, connecting VE-Cad/ β-catenin/TCF-4 expression with the induction of VM in metastatic melanoma cells. Specific inhibitors of FAK activity (Y397) abolish the formation of VM through a novel mechanism involving the dissociation of β-catenin from VE-Cadherin and allowing the inactivation of TCF-4-dependent gene expression. Remarkably, concomitant inhibition of VM (using a specific FAK inhibitor) with “classical” anti-angiogenesis treatment with bevacizumab, resulted in the improved control of uveal melanoma in vivo tumor growth.

## Results

### LC-MS approach showed an association between β-catenin and VE-cadherin in VM prone uveal melanoma cells

Non-endothelial VE-Cadherin expression has been examined in many different cancer types associated with VM. Although the expression of VE-Cadherin (CD144 or CDH5) has been correlated with an endothelial context, its role in the transformation of tumor cells has received increasing attention in the last decades. We recently reported that the VM-prone cells express Y658 of VE-Cad through the intrinsically active FAK [14], which correlates upstream with the basal mutation of GNAQ/GNAQ11 in the rare cancer uveal melanoma [3].

Since the first description of β-catenin/VE-cadherin in 1995 [15], a vast scientific literature has revealed the vital importance of these complexes in adherent junctions, cell permeability [16, 17], leukocyte infiltration [18], epigenetic alterations [19] in the context of endothelial cells [20], but the implications in VM of these complex have not been explored yet.

**Fig. 1:**
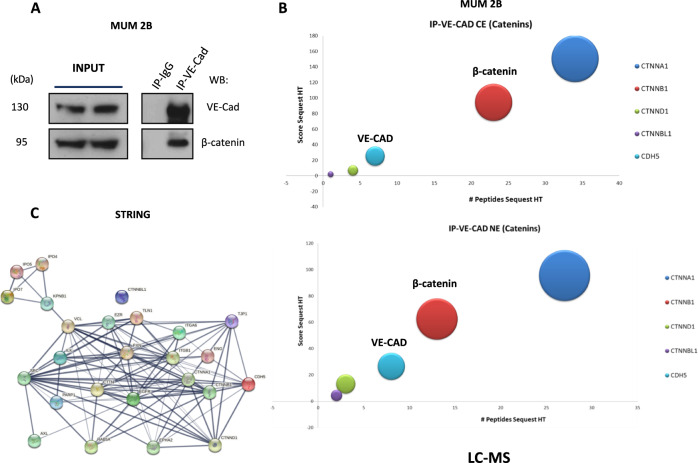
LC-MS experiments showed an elevated coupling of β-catenin/VE-cadherin complex in VM. **A** Immunoprecipitation of VE-cadherin experiments shows union with β-catenin in MUM 2B cells, **B** representative union catenin to VE-cadherin graph of LC-MS proteomics IP-VE-cadherin CE (cytosol extract), NE (nuclear extract), score sequest HT in the X axis and #peptides sequest HT in *Y* axis. **C** String graph union protein of VE-cadherin.

To get more specific information about the consequences of the anomalous expression of VE-cadherin in the nucleus in VM prone cells we performed a proteomic analysis to identify VE-cadherin-interacting proteins. Proteomic analysis of proteins complexes to VE-cadherin using Liquid Chromatographic-Mass Spectrometry (LC-MS) in a cytosol-nucleus subfractionation (Tables S1, S2) showed that β-catenin was one of the most represented proteins in complex with VE-Cadherin in both compartments in MUM 2B cells (Fig. 1B) and represented the most frequent protein complex by STRING analysis (Fig. 1C). The presence of a VE-Cadherin/β-catenin complex was further corroborated by co-immunoprecipitation assay of VE-cadherin in MUM 2B cells Fig. 1A, Fig. S1D and HUVEC cells Fig. S1E.

### Elevated expression of active β-catenin and Y397 phosphorylation of FAK in aggressive melanoma cells

**Fig. 2:**
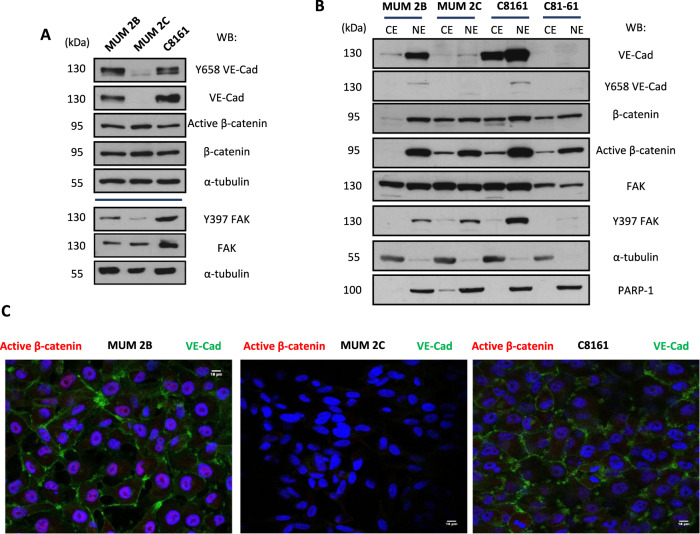
Elevated expression of active β-catenin and Y397 phosphorylation of FAK in aggressive melanoma cells. **A** Total expression of VE-cadherin, Y658 VE-cadherin, of β-catenin or non-phospho β-catenin (active β-catenin), focal adhesion kinase (FAK) or the active form phospho FAK (Y397) were measured in aggressive melanoma cells (MUM 2B, C8161) and poorly melanoma cells (MUM 2C, C81-61), and **B** in a subfractionation experiment. **C** We confirm these results in an immunofluorescent experiment (active β-catenin: red, VE-cadherin: green and DAPI (nuclear stain, blue). Bars 15 μm. **D** Immunochemistry analysis is performed to observe nuclear Y658 VE-Cadherin expression in melanoma in situ, metastasis patients. Consecutive sections are shown. Bars: 58 μm.

To confirm the association between β-catenin/FAK/VE-Cadherin identified by LC/MS, we performed a group of experiments aiming to analyze the status of these proteins in VM prone cells. Total expression of β-catenin or non-phospho β-catenin (active β-catenin), focal adhesion kinase (FAK) or the active form phospho FAK (Y397) were measured in different melanoma cell lines from either uveal melanoma cells: MUM 2B (VE-Cadherin positive, NRP-1 positive, VEGFR2 negative, Fig. S1A) and MUM 2C (VE-Cadherin negative, NRP-1 negative, VEGFR2 negative, Fig. S1A), or cutaneous melanoma cells: C8161 (VE-Cadherin positive, NRP-1 positive) and C81-61 (VE-Cadherin negative, NRP-1 negative). MUM 2B and C8161 have high expression of phospho-FAK compared with MUM 2C (Fig. 2A). We performed cytosol-nucleus subfractionation experiments in these cells, showing that MUM2B and C8161 cells display an increased expression of active β-catenin in the nuclear compartment, (Fig. 2B). We corroborate these results by indirect immunofluorescence (Fig. 2C), which confirms the nuclear localization of active β-catenin in VE-Cadherin positive cells (MUM 2B and C8161). To confirm this nuclear VE-cadherin expression, immunohistochemistry experiments from cutaneous melanoma patients confirm the nuclear expression of Y658 VE-Cadherin focalized in the metastasis situation of the tumor represented in Fig. S3C. We also validated the subcellular localization of VE-Cad, Y658 and possible contamination by the heavy membrane of subfractionation experiments in Fig. S1B. We performed a 3D angiogenesis assay in MUM 2B-GFP cells in matrigel to confirm the tubular-like network produced by VM cells (Fig. S1C).

### VE-Cadherin/β-catenin forms a complex in a FAK-dependent manner leading to enhanced TCF-4 transcription activity

**Fig. 3:**
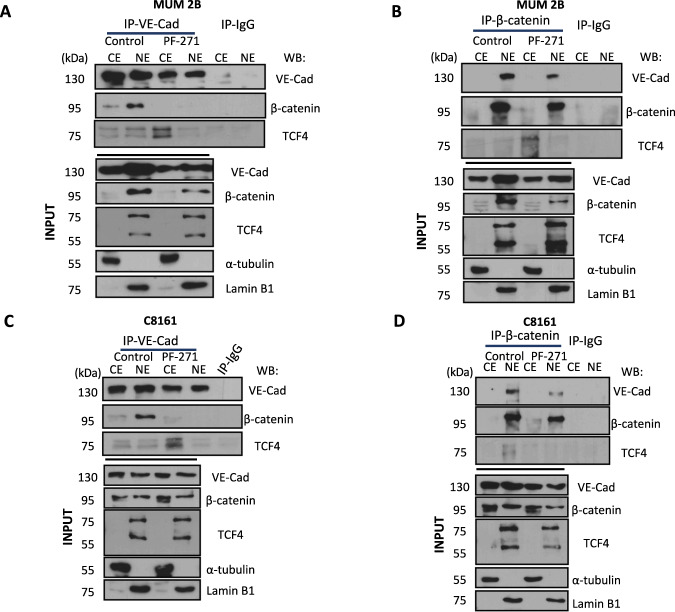
VE-Cadherin/β-catenin forms a complex in FAK-dependent manner to enhance TCF-4 transcription activity. **A** Immunoprecipitation after cytosol-nucleus fractionation of VE-Cadherin or β-catenin **B** in MUM 2B cells, input was used to protein expression controls, α-tubulin cytosol control or lamin B1 nucleus control. **C** Immunoprecipitation after cytosol-nucleus fractionation of VE-Cadherin or β-catenin **D** in C8161 cells, input was used for protein expression controls, α-tubulin cytosol control or lamin B1 nucleus control.

Our group has recently reported that human malignant melanoma cells with the capacity to form VM have an intrinsically high expression of pVE-cadherin at Y658. VE-cadherin is a substrate of focal adhesion kinase (FAK) and forms a complex with p120-catenin and the transcriptional repressor Kaiso or ZBTB33 in the nucleus of the cells. Immunoprecipitation experiments of VE-Cadherin and β-catenin were performed in the absence/presence of the FAK inhibitor PF-271 (Fig. 3A, B) or after siFAK (Fig. S2A); PF-271 is a potent competitive inhibitor that specifically affects the phosphorylation of FAK, but not its stability; more specifically, PF-271 affects the Y397 residue of FAK (Fig. S2C) independently of the possible activity of inhibitor on the phosphorylation of Src Y416 [21] and affect the stabilization of YAP through p-S127 of YAP [7] overexpression with other FAKi as well (PND-1186) (Fig. S2C) in MUM 2B cells and C8161 cells (Fig. 3C, D) exhibited that β-catenin need the phosphorylation Y658 of VE-Cadherin to form this complex, even more, with the β-catenin direct transcription factor TCF-4 in a FAK dependent manner.

**Fig. 4:**
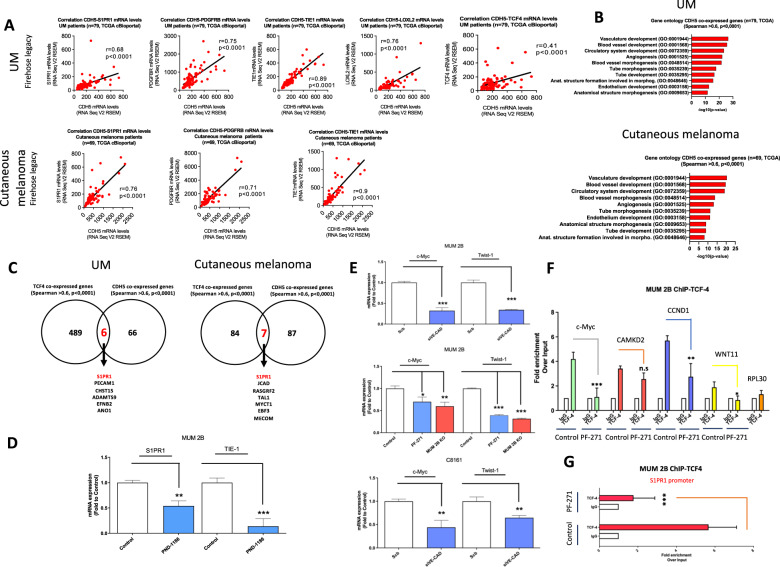
VE-Cadherin/β-catenin forms a complex in FAK-dependent manner enhance TCF-4 transcription activity. **A** Evaluation of the correlation between CDH5 and S1PR1, PDGFRB, TIE1, LOXL2, and TCF4 mRNA levels in a cohort of uveal melanoma (*n* = 79, TCGA Firehose legacy) and cutaneous melanoma patients (*n* = 69, TCGA, Firehose legacy) using cBioPortal. Correlation analysis was done using the Spearman test. **B** Top 10 gene ontology terms of CDH5 positive correlated genes (*r* > 0.6, *p* < 0.0001) from previous cohorts. **C** Venn diagram comparing CDH5 and TCF4 positive correlated genes was done using the Spearman test (*r* > 0.6, *p* < 0.0001). **D**, **E** qPCR experiments with FAK-inhibitors, silencing VE-Cadherin (50 nM during 48 h) in MUM 2B and C8161 cells or MUM 2B ko cells showed strong downregulation of TCF-4-dependent genes (c-Myc, Twist1, S1PR1) and VM implications gen TIE-1. Statistical analyses were conducted using Graph Pad Prism software. Statistical significance was calculated using a Student’s t-test (unpaired, two-tailed) with measurements from at least three independent trials. **F** ChIP-TCF4 assay in MUM 2B cells at the Wnt11 promoter, CCND1 promoter, c-Myc promoter, and S1PR1 with and without PF-271 treatment. CAMK2D promoter is used as a positive control and RPL30 as an irrelevant sequence for TCF-4 binding (negative control). Results are represented as fold enrichment over input. Asterisks denote significance in an unpaired t-test (*p* < 0.05, *p* < 0.01, *p* < 0.001), and error bars denote SD. **G** ChIP-TCF4 assay in MUM 2B cells at the S1PR1 promoter with and without PF-271 treatment.

After a comprehensive analysis from the cBioPortal database, a platform of 48,333 tumor samples, we found that CDH5 gene expression highly correlated with numerous alterations leading to VM as well as angiogenesis development in uveal melanoma database (*N* = 79, TCGA) and cutaneous melanoma (*N* = 69, TCGA). Some of the alterations identified are drivers of different tumors and for VM formation: S1PR1 (breast cancer [22]), PDGFRB, TIE-1 (melanoma [5, 23]), LOXL2 (hepatocellular carcinoma [24]) and interestingly TCF-4 (Fig. 4A). Moreover, we used a web database of Gene Ontology with the 10 genes significantly altered and showed that CDH5 co-expressed genes majority correlate with angiogenesis pathways, revealing the pivotal role of CDH5 in the formation of VM in uveal melanoma as well as cutaneous melanoma (Fig. 4B). Using a Venn diagram with TCF-4 co-expressed genes versus CDH5 co-expressed genes, 7 genes were robustly correlated, being S1PR1 the most prominent one not only in uveal melanoma but also in cutaneous melanoma (Fig. 4C). We confirm the implication of Y658 phosphorylation in VM formation, through FAK activity inhibition (PND-1186) in qPCR experiments, showing a strong reduction of S1PR1 and TIE-1 mRNA levels as wells as protein expression (Figs. 4D and S2E).

Wnt pathway-dependent gene expression is transduced by the nuclear effector β-catenin. Due to the lack of a DNA-binding domain, β-catenin binds indirectly Wnt-responsive genes by interacting with TCF/LEF family members [25].

After the decoupling of the β-catenin/VE-Cadherin complex following VE-Cadherin silencing (Fig. 4E), the absence of VE-cadherin (MUM 2B VE-Cad KO cells) and FAK inhibition (Fig. 4E) lead to the down-regulation in the expression of TCF-4-dependent genes (c-Myc and Twist-1) as determined by qPCR experiments and western blot Fig. S2E. The qPCR results were corroborated in C8161 melanoma cells after VE-Cadherin silencing experiments (Fig. 4E).

We further examined the effect of preventing VE-Cadherin phosphorylation in the recruitment of TCF-4 to target promoters by performing chromatin IP. FAKi treatment robustly decreased TCF-4 binding to the CCND1 and C-myc promoters and induced a moderate decrease of WNT-11 (Fig. 4F) and S1PR1 promoters (Fig. 4G), in concordance with the TCGA analysis in UM and cutaneous melanoma patients (Fig. 4C). We used CAMK2D and RPL30 sequences as positive and negative promoter sites of TCF-4 respectively (Fig. 4F).

### Abrogation of VM formation through siRNA of TCF-4 target genes and β-catenin silencing/TNKs inhibitors

**Fig. 5:**
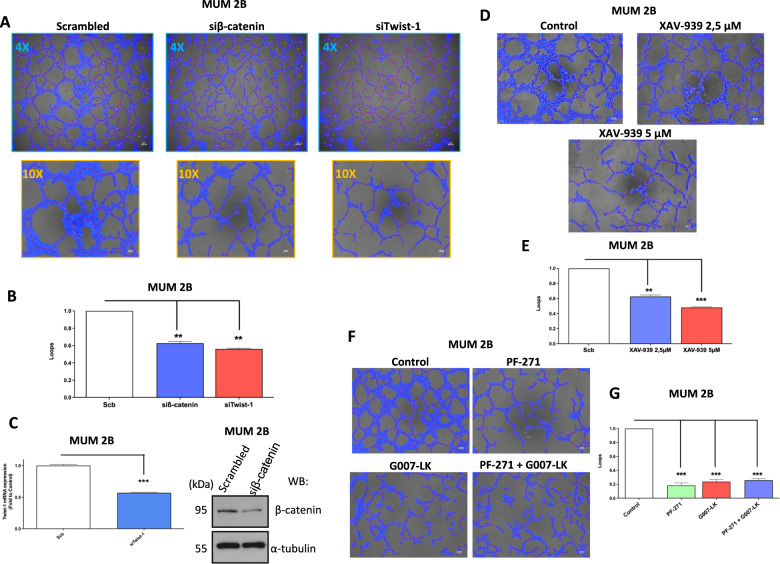
Abrogation of VM through siRNA of TCF-4 target genes and β-catenin silencing/TNKs inhibitors. **A** In vitro angiogenesis assay with Matrigel in MUM 2B showed the effect of siβ-catenin or siTwist1, images were acquired using an Olympus CKX41 microscope (10× or 4× lens) (bars 50μm) and the formation of tube-like structures was then quantified by Wimasis program. **B** Each treatment was performed in triplicate, and the experiment was independently repeated at least three times. **C** siRNA of Twist1 was confirmed by qPCR and siβ-catenin by western blot experiments. **D** Angiogenesis assay with TNKS inhibitor (XAV-939 2,5 µM and 5 µM during 24 h) and quantification by wimasis program represented in **E** Bars: 100 µm. **F** In vitro angiogenesis assay with Matrigel in MUM 2B showed the effect of PF-271 (1 µM during 24 h) or G007-LK (5 µM during 24 h), images were acquired using an Olympus CKX41 microscope (10× lens) (bars 50 μm) and the formation of tube-like structures was then quantified by **G** Wimasis program. Each treatment was performed in triplicate, and the experiment was independently repeated at least three times. Results are represented as fold enrichment. Asterisks denote significance in an unpaired t-test (*p* < 0.05, *p* < 0.01, *p* < 0.001), and error bars denote SD.

Because of the elevated expression of FAK/Y397 in uveal melanoma samples with predominantly high levels of pVEC (Y658) by malignant melanoma cells, we investigated the cell’s capacity to generate de novo vasculogenic-like network in a 3D matrix (Matrigel) after the alteration of the upstream of TCF-4 target genes (Twist-1) and directly attack to β-catenin; results in Fig. 5A show that siTwist-1 or siβ-catenin abolished VM formation as determined by the capacity to form loops quantified by Wimasis (Fig. 5B); siTwist-1 and siβ-catenin were confirmed by qPCR and western blot respectively (Figs. 5C and S2F). Tankyrases (TNKS) proteins belong to the Poly (ADP-ribose) polymerases (PARPs) family and TNKS1 has been the best shown to be the most relevant and abundant of the two isoforms. TNKSs are implicated in diverse functions such as telomere elongation [26], and Wnt/β-catenin signaling [27] among others. As has been explained above, β-catenin plays a significant role in Wnt signaling and is tightly adjusted by the β-catenin destruction complex, which is composed of APC, CK1α, GSK3β and Axin proteins. This complex is intrinsically involved in the phosphorylation of β-catenin, which then is identified and ubiquitinated by the ubiquitin ligase SCFβ-TrCP to carry out its degradation for proteasome complex [28, 29]. Recently, our group reported that p120 catenin protects the stability of VE-cadherin in VM prone melanoma cells [30]. In the current study, we decided to perform a similar experiment after silencing β-catenin, and show the same results as after silencing p120 in MUM 2B cells (Fig. S2B), demonstrating the importance of the stability of proteins anchored to VE-Cadherin. Because of the pivotal role of β-catenin on the stability of VE-cadherin, angiogenesis assay showed after inhibition of TNKs (as a way to down-regulated β-catenin levels) with different concentrations (XAV-939) abolish the capacity to form VM in uveal melanoma cells (Fig. 5D) and quantified by Wimasis program (Fig. 5E). Consequently, we decided to perform the same in vitro VM experiments with a different inhibitor of TNKs (G007-LK, 5 μM during 24 h) in the presence or absence of inhibitor of FAK (PF-271, 1 μM during 24 h). Results in Fig. 5F, G, show an important decrease of VM formation in both treatments, and we corroborated the effect of TNKs inhibitor from β-catenin by western blotting after G007-LK treatment represented in Fig. S2E.

### VE-cadherin (Y658) is crucial in the ability to form VM by stabilization of β-catenin and TCF-4 gene transcription

**Fig. 6:**
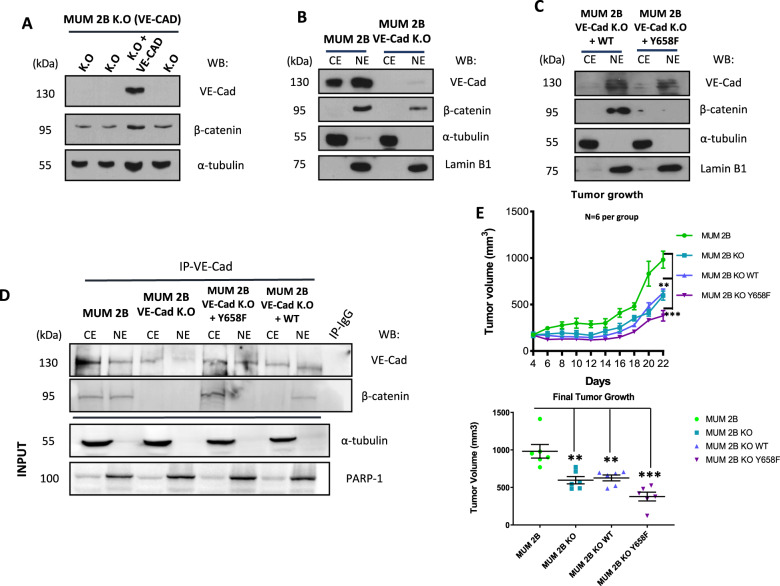
Y658 phosphorylation of VE-cadherin is essential in the ability to form VM by stabilization of β-catenin and TCF-4 gene transcription. **A**–**C** Western blot assay, VE-Cad K.O, VE-Cad K.O WT VE-Cad, VE-Cad K.O Y658F construct (1µgr during 48 h) in MUM 2B showed that VE-cadherin/Y658 is essential for β-catenin stabilization. **D** Immunoprecipitation after cytosol-nucleus fractionation of VE-Cadherin in MUM 2B cells, VE-Cad K.O, VE-Cad K.O WT VE-Cad, VE-Cad K.O Y658F construct (1 µgr during 48 h), input was used to protein expression controls, α-tubulin cytosol control or PARP-1 nucleus control. **E** Tumor growth progression graphic representation on different days, the annotations of the tumor were every two days, being the day 4 post-injection of MUM 2B cells, VE-Cad K.O, VE-Cad K.O WT VE-Cad, VE-Cad K.O Y658F construct (1 µgr during 48 h) before the beginning the injection of the cells (*N* = 6 per group). Results are represented with asterisks that denote significance in an unpaired t-test (*p* < 0.05, *p* < 0.01, *p* < 0.001), and error bars denote SD.

To further substantiate the impact of the phosphorylation of Y658 of VE-Cadherin in the capacity to form VM and β-catenin stability, we performed a cytosol-nucleus subfractionation experiment in MUM 2B cells permanently knock-out for VE-Cad after gene editing with CRISPR/Cas9, which were rescued with either VE-Cadherin WT or the mutant/non-phosphorylable VE-Cadherin (Y658F) (Fig. 6A–C). The re-introduction of VE-Cad in knock-out cells with VE-Cadherin WT increased β-catenin expression (Fig. 6A), contrary to what was observed in MUM 2B KO that abolish the global β-catenin expression, as well as after the re-introduction of the mutant VE-Cadherin (Y658F) in VE-Cadherin knock-out cells (Fig. 6C). To confirm the pivotal role of Y658 phosphorylation of VE-Cadherin in the trafficking and stability of β-catenin, we performed a VE-Cadherin co-immunoprecipitation experiments in MUM 2B, MUM 2B KO, MUM 2B KO WT (VE-Cad rescue) and MUM 2B KO Y658F (non-phosphorylable VE-Cad), showed that the β-catenin union only occur in cytosol compartment in the Y658F conditions, contrary to MUM 2B KO VE-Cad rescue (WT) (Fig. 6D). Finally, to further explore the role of the β-catenin/VE-cadherin complex in tumor progression in metastatic uveal melanoma cells, a MUM 2B xenograft nude mice experiment showed that MUM 2B KO Y658F reduced considerably tumor growth (Fig. 6E), suggesting an essential role of VM and VE-Cad/ β-catenin axis in tumor progression. Overall, these results suggest that Y658 phosphorylation of VE-Cadherin is essential in the stability of β-catenin in aggressive melanoma cells and consequently in the formation of VM.

### FAK inhibition in combination with prime line anti-angiogenesis Bevacizumab reduces the tumor growth in MUM 2B xenograft approach

**Fig. 7:**
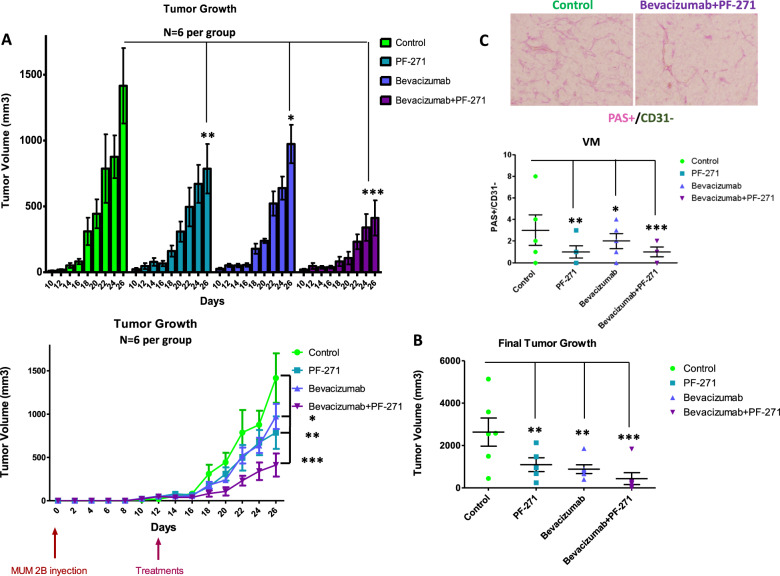
FAK inhibition in combination prime line anti-angiogenesis Bevacizumab reduces the tumor growth in MUM 2B xenograft approach. **A** Tumor growth progression graphic representation on different days, the annotations of the tumor were every two days, being the day 12 post-injection of MUM 2B cells the beginning of different treatments. **B** Graphic representing final tumor growth with the treatment’s groups (*N* = 6). **C** Representative pictures of different tumors from treatment groups.

FAK inhibition has been shown to reduce melanoma [31], breast [32], and ovarian [33] tumor metastasis. Anti-angiogenic therapies against tumor progression (as is the case for antibodies anti-VEGF receptor bevacizumab) have had limited results so far in different cancer settings [34–36]. Therefore, the development of novel anti-tumor neovascularization strategies to treat cancer cells is of essential importance; the challenge remains to enlarge the targets from prevailing angiogenesis to all the alternative mechanisms of aberrant angiogenesis recently reported, such as VM [37]. Targeting VM with specific small molecule compounds combined with front-line therapies may represent the best approach to obtaining a good anti-tumor response in patients. With this aim, we set up a xenograft approach with MUM 2B cells in which we combined the prime line FDA-approved to clinic-used anti-angiogenesis treatment Avastin/Bevacizumab plus a potential specific inhibitor of Y397 phosphorylation, PF-271 (represented in Fig. 7A), as a way to limit VM, according to us in vitro results. Tumor growth was evaluated 26 days after the inoculation of MUM2B in nude mice (*N* = 6 per group), the combination of Bevacizumab plus PF-271 reduced global tumor progression, as well as the final tumor growth **(**Fig. 7B, Fig. S3). The reduction of PAS+/CD31− (as a surrogate of VM regions) in tumors after the new anti-angiogenesis approach treatment is represented in Fig. 7C. Interestingly, PF-271 plus Bevacizumab treatment also increases apoptosis and decreases cell proliferation as derived from direct microscopy morphological evaluation of IHC (presented in Table S6).

## Discussion

Anti-angiogenic therapies have been developed against VEGF anchoring on endothelial cells (bevacizumab, approved by the FDA in 2004 for the treatment of colorectal cancer), [38]. Although encouraging, positive patient responses were rarely seen in early clinical trials. The clinical advance for anti-angiogenic therapy emerged from a phase III trial that evidenced substantially [39] prolonged survival when bevacizumab, was concomitantly applied with chemotherapy in patients with metastatic colorectal cancer [40]. Based on these reports, bevacizumab became the first anti-angiogenic agent to be authorized by the US Food and Drug Administration (FDA) for the treatment of cancer. In successive phase III trials, bevacizumab in unification with standard chemotherapy improved overall survival in lung cancer patients and disease-free survival in breast cancer patients [41]. Additionally, it has been described to be active in patients with metastatic renal cell cancer as monotherapy (enhanced disease-free survival, but not overall survival) [42]. A possible cause of the inconsistency of these results related to the use of anti-angiogenic agents may be due to the presence of VM phenomena produced by the tumor cells themselves.

Aberrant microcirculation, including VM, is a major driving factor for the formation of hypoxic areas and treatment failure in solid tumors by the aggressiveness and complex tumor microenvironment is mainly determined by an aberrant tumor microcirculation. VM is the predominant blood supply in early malignant melanoma tumor growth; prevailing endothelial-based vessels are assisted by VM in the later stages of tumor expansion [43]. In addition to promoting growth, VM also provides to enlarge the portion of tumor cells that are located adjacent to blood flow, enhancing their chance of infiltrating the bloodstream and metastasizing to distant locations in the body [44]. Therefore, the existence of VM is linked with distant metastases, poor overall survival, and local cancer relapse. Extra-vascular expression of VE-cadherin has been studied in several cancer types associated with VM [45]. Our group has reported that human aggressive melanoma cells have a constitutively high expression of pVE-cadherin. pVE-cadherin Y658 is a substrate of focal adhesion kinase (FAK) and forms a complex with p120-catenin and the transcriptional repressor Kaiso in the nucleus. Interestingly, uveal melanoma cells genetically deficient for VE-cadherin lost the ability to develop VM [14]. The identification of a new key factor in VM development, β-catenin/TCF-4, would enable the introduction of a new treatment approach to reduce tumor growth due to the reduction of the ability to form VM in uveal melanoma cells that have previously been shown to have a mutation in GNAQ proteins (leading to constitutive FAK activation through YAP), which leaves them exposed to perform a targeted treatment in rare cancer as well as cutaneous melanoma. In the present study, we have shown that FAK plays an important role in the re-localization of the VE-Cad/β-catenin complex, associated with phosphorylation of Y658 of VE-Cadherin and the pivotal role in the Vasculogenic Mimicry formation (aberrant angiogenesis production).

**Fig. 8:**
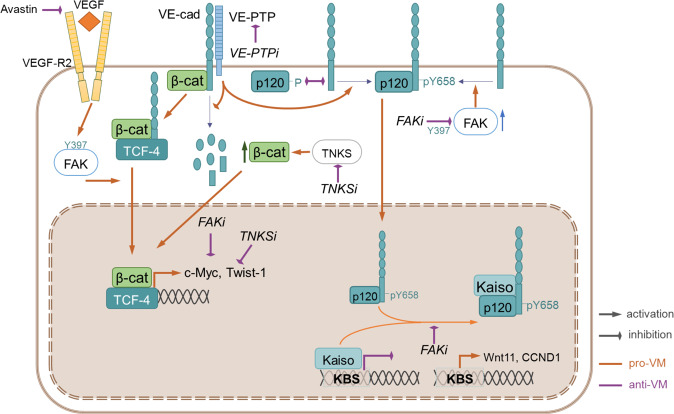
Proposed model for the mechanism by which elevated Y658 of VE-Cadherin/ β-catenin/TCF-4 axis leads to increased VM in malignant melanoma cells. VM VE-Cadherin positive cells express a basal activate FAK Y397 enhance Y658 VE-Cadherin implication to different transcription factors (Kaiso, TCF-4) as well as target genes (CCDN1, Wnt11, C-Myc, Twist-1), VEGFi, FAKi, TNKSi, and VE-PTPi finally inhibit VM formation in cutaneous/uveal melanoma cells. Further details are explained in the “Discussion” section.

FAKi prevents nuclear relocation of the complex and allows, in the context of VM formation during tumor development, to enhance TCF-4 activity by boosting the expression of TCF-4-dependent genes. For instance, silencing of β-catenin produces an important reduction of VE-Cadherin, suggesting that β-catenin is involved in its degradation probably by autophagy; about this degradation process, we have shown in a previous study [30] that the binding of p120 (another important catenin involved in adherent junctions) to VE-Cadherin has an important role in the autophagy-dependent degradation of VE-Cadherin. Conceptually, the presence of VE-cadherin in the nucleus remains challenging. Recently, a proteomic seminal approach reported by Adam Bryon et al. [46] showed that some adhesion proteins like integrin and others involved in adherents junctions have nuclear localization and are called nucleo-adhesome regions nearly to the nucleus. More interestingly, the proteins associated with nucleo-adhesome are FAK-dependent way. Moreover, it has been reported by different groups [47] that a small amount of cadherin (E-cadherin) can be transported to the nucleus either as a short-length [48, 49] (or cleaved) of the protein or as full length [50] by cancer cells in acetylation way, and consequently affected the β-catenin transcription activity, may represent an excellent approach to see the possible implications of VE-Cadherin acetylation in a VM context (Fig. 8). In recent years, several clinical trials have been initiated that are in Phase I and II with FAK inhibitors; however, some of FAK’s roles in tumorigenesis remain under investigation [33, 51]. Targeting a single VM cell population [52, 53] with specific target compounds (FAKi) combined with anti-angiogenesis front-line therapies may represent the best approach to acquiring a good prognosis in patients in the present-future. Thus, pharmacological inhibition of FAK activity with PF-271 or similar small molecule inhibitors will impact in Y658 VE-cadherin with important consequences in beta-catenin-derived TCF4/LEF activation of gene expression and may imply a new therapeutic opening in the overall repression of genes involved VM promotion in cancer cells. In conclusion, we demonstrate that VE-Cadherin in partnership with FAK activity and β-catenin act as sensors of tumor microenvironment and under unfavorable conditions opt to form VM to allow nutrient and oxygen attraction by cancer cells. Thus, targeting this axis might be an alternative to overcome the resistance of tumors to classic anti-angiogenic approaches.

## Materials and methods

### Reagents and antibodies

The following reagents were used: Avastin/Bevacizumab (Roche) 5 mg/Kg I.P two days per week, PF-562271 (Selleckchem) 1 μM during 24 h in vitro assays and 30 mg/Kg oral gavage every two days in tumor xenograft assay, PND-1186 (Selleckchem) 1 μM during 24 h in vitro assays, XAV-939 2.5-5 μM during 24 h and G007-LK 5 μM during 24 h. Corning Matrigel Basement Membrane Matrix for in vitro angiogenesis experiments. Antibodies used were: Y658 VEC rabbit (1:1000 WB, 1:100 IF, Thermofisher), VEC C-ter mouse (1:500 WB, 1:50 IF, 2 µg IP, clone F-8, sc-9989), β-catenin mouse (1:1000 WB, 2 µg IP, sc-7963), non-phopho β-catenin mouse (1:1000 WB, 1:100 IF, Clone D13A1, cell signaling), phopho Y397 FAK rabbit (1:1000 WB, Clone 44-624G, Thermofisher), FAK rabiit (1:1000 WB, Clone C-20, sc-558), TCF-4/TCF7L2 rabbit (1:1000 WB, Clone C48H11, cell signaling), TNKS mouse (1:1000 WB, Clone E-10, sc-365897), phopho-YAP S127 rabbit (1:1000 WB, Clone 4911, cell signaling), YAP rabbit (1:1000 WB, Clone D8H1X, cell signaling), α-tubulin mouse (1:10,000 WB, clone B-5-1-2, Sigma-Aldrich), lamin B1 rabbit (1:1000 WB, Abcam) and PARP-1 mouse (1:1000 WB, Calbiochem), Twist-1 rabbit (1:1000 WB, E7E2G, cell signaling), c-Myc mouse (1:1000 WB,), TIE-1 rabbit (1:1000 WB, D2K2T, cell signaling).

### Cell lines and construction of GFP-tagged VEC

Human melanoma cells MUM 2B (GNAQ mutant) [54, 55], C8161, MUM 2C and C81-61 were grown in RPMI medium supplemented with 10% fetal bovine serum, 2 mM of L-glutamine, and 1% penicillin/streptomycin (PAA laboratories). Human umbilical vein endothelial cells (HUVEC) were grown in endothelial cells growth medium-2 (EGM-2) (Lonza). All cells were cultured at 37 °C and 5% CO_2_ in incubator cells. cDNA of human VEC fused in-frame with GFP at the COOH-terminus (VEC-GFP), VEC Y658F was a kind of gift from Dr Masahiro Murakami. These constructs were subcloned into pcDNA3.1 (Invitrogen), and previously validate [14].

### In vitro angiogenesis assay

The effect of PF-271, XAV-939, and G007-LK after 24 h and siβ-catenin, siTWIST-1 after 48 h on the formation of tube-like structures in Matrigel (BD Biosciences) was resolved according to the manufacturer’s instructions and previously describe [30]. After 48 h, respectively, of incubation, images were acquired using an Olympus CKX41 microscope (4× and 10× lenses). The formation of a tube-like organization was quantified by the Wimasis program. Each treatment was realized in triplicate, and the experiments were independently repeated at least three times.

### Quantitative RT-PCR

Total RNA was isolated by RNeasy Mini Kit (Qiagen) according to the manufacturer’s recommendations and previously described [14]. Each reaction was performed in triplicate using CFX96 Real-time PCR detection systems. Primer sequences for the targets and the annealing temperature (60 °C): 36B4: Forward 5′-CAGATTGGCTACCCAACTGTT-3′, Reverse 5′-GGCCAGGACTCGTTTGTACC-3, c-Myc: Forward 5′-AAAGGCCCCCAAGGTAGTTA-3′, Reverse 5′-GCACAAGAGTTCCGTAGCTG-3′; Twist1: Forward 5′-GCAGGACGTGTCCAGCTC-3′, Reverse 5′- CTGGCTCTTCCTCGCTGTT -3′, S1PR1: Forward 5′- F: 5’-CTCGAGTAAGTTTGCGAGAG-3′, Reverse 5′- TGGTTCGATGAGTGATCCAG3′, TIE-1: Forward 5′- CAAGGTCACACACACGGTGAA-3′, Reverse 5′- GCCAGTCTAGGGTATTGAAGTAGGA -3′.NRP-1: Forward 5′-AAAACGGTGCCATCCCT-3′, Reverse 5′- AAGAAGCAGAGTGGGTCGTT -3′.

### Gene editing

MUM2B knockout (ko) cells for the VE-Cad gene were composed using the CRISPR-Cas9 technology. Five different sgRNAs were designed using the Zhang Lab Optimized CRISPR design tool and cloned into the pL-CRISPR.EFS.GFP which was purchased from the Addgene public repository (#57818) and previously describe [14].

### Transfection of small interfering siRNA

Cultured cells were transiently transfected with an irrelevant siRNA: 5′- CCUACAUCCCGAUCGAUGAUG-3′, siVE-Cad: 5′- AGAUGCAGAGGCUCAUGAUTT -3′ 50 nM, siβ-catenin: 5′-CAGGGGGUUGUGGUUAAGCUCUU-3′ 50 nM, siTwist1: 5′- AAGCTGAGCAAGATTCAGACC-3′ 50 nM and siFAK: 5′-GGUUCAAGCUGGAUUAUUU-3′ 50 nM were transfected for 48 h using JetPrime (Polyplus transfection) according to the recommendations.

### Chromatin Immunoprecipitation (ChIP)

Cells were grown to approximately 80–90% (18 × 10^6^ cells per IP) confluence with PF-271 1 µM for 24 h with the respective controls. The culture medium was aspirated and cells were washed twice with cold PBS. ChIP was performed following SimpleChIP Enzymatic Chromatin IP Kit (Magnetics beads) (Cell signaling). qPCR promoter specific primers used: c-Myc promoter, Forward 5′-GCTCAGTCTTTGCCCCTTTGTGG-3′, Reverse 5′- TAACACCTTCCCGATTCCCAAGTG-3′; CCND1 promoter [56], Forward 5′-TTTACATCTGCTTAAGTTTGCG-3′, Reverse 5′- TTAGAATTTGCCCTGGGACT-3′; WNT11 promoter, Forward 5′-CACCCTTCCCACTTCCAA-3′, Reverse 5′-GAGAGACGTCTGCTTGGCT-3′ and S1PR1 promoter Forward: 5′-GGGGTACCGCCTCTTCCTGAAGAA-3′,Reverse:5′-CCAAGCTTCTCGCAAACTTACTCG-3′, CaMK2D gene promoter as a positive control of ChIP-TCF4 from cell signaling (Human CaMK2D Intron 3 #5111) and negative control RPL30 with irrelevant sequence.

### Immunoblotting, immunoprecipitation, subfractionation cytosol-nucleus

Simple coimmunoprecipitation, cells were lysed in lysis buffer (50 mM Tris/HCl ph 8, 120 mM NaCl, 0.1% NP-40, 1 mM EDTA, 10 mM NaF, 1 mM Na_3_VO_4_ and supplemented with a protease inhibitor cocktail (1 tablet to 10 ml of lysis buffer, Roche) for 30 min at 4 °C and previously describe [14].

Accordingly with the article of [57], for subfractionation cytosol-nucleus, cells were lysed in lysis buffer (250 mM sucrose, 50 mM Tris-HCl ph 7,4, 5 mM MgCl_2_, 1 mM Na3VO_4_, 0.25% NP-40 and supplemented with a protease inhibitor cocktail (1 tablet to 10 ml of lysis buffer, Roche) for 10 min at 4 °C.

### In vitro 3D angiogenesis assay

Tube-like 3D structures in Matrigel (BD Biosciences) were calculated according to the protocol from [58]. Images of MUM 2B-GFP were obtained in the linear range of detection to avoid signal saturation using a fluorescent microscope confocal microscopy (Leica SP5, 63× lens) with swept Z-stack.

### LC-MS/gene enrichment analysis

Cells were resuspended in 60 μl after IP-CE, NE subfractionation, were precipitated with acetone and resuspended in 50 mM ammonium bicarbonate solution containing 8 M urea. After reduction and alkylation, samples were digested with trypsin and desalted with Clean-Up. Enriched samples were analyzed on nano-LC-MS in Easy-nLC 1000 (Proxeon) coupled to an ionic source with nano electrospray (ThermoScientific). Raw files were analyzed in the Uniprot database using Sequest in Proteome Discoverer (ThermoScientific) (Proteomic department from UCO). Peptide identification was validated by Percolator using *q* value ≤ 0.01.

Gene ontology terms for each gene were obtained from the Bioconductor Homo Sapiens database and related to Entrez gene identifiers in an orgDB R object through the annotation forge package to be used with the cluster profiler. Thus, Gene ontology enrichment analysis was acquired for biological processes, molecular functions and cellular complex terms (REVIGO) (Fig. S4). The list of select proteins (Table S3) was analyzed in the STRING database to define the interaction. Default parameters were used to identify significantly enriched gene sets (FDR *q* < 0.25). Non-specific adjustment IP-IgG was measured as a control in LC-MS experiments (Tables S4, S5).

### Tumor Xenograft assay

Male Swiss Nude (SwN) mice were purchased from Charles River Laboratories and housed at IPBLN-CSIC and IBIS animal facility according to institutional guidelines (Approved Ethical Committee). For xenograft generation, 2 × 10^6^ MUM 2B cells, MUM 2B KO, MUM 2B KO WT, and MUM 2B KO Y658F in 100 µL PBS were subcutaneously injected in the flank of 7-week-old mice. Animals (*n* = 6 per group) were monitored every two days after cell injection until the final time point when they were sacrificed and tumors were dissected for further analyses. Tumor volume was calculated as: in progress tumor volume = (π × length × width^2^)/6 and final tumor volume = (π × length × width × height)/6).

### Immunofluorescence

Immunofluorescence was performed on cells plated onto coverslips and grown for 24 h before experimental treatment. The cultured media was removed and washed two times with PBS 1× and the cells were fixed (3% Paraformaldehyde, 5% sucrose) for 15 min at room temperature. Permeabilization was handled using 0.25% Triton-X100 in PBS for 10 min and previously describe [45]. Immunofluorescence images were acquired in the linear range of detection to avoid signal saturation using a fluorescent microscope confocal microscopy (Leica SP5, 63× lens).

### Immunohistochemistry

Four-micrometer-thick tissue sections from paraffin blocks were dewaxed in xylene and rehydrated in a series of graded alcohols. Sections were immersed in 3% H_2_O_2_ aqueous solution for 30 min to exhaust endogenous peroxidase activity, then covered with 1% blocking reagent (Roche, Mannheim, Germany) in 0.05% Tween 20-PBS, to block nonspecific binding sites. Sections were incubated with primary antibodies for 1 h for anti-Y658 VE-Cadherin (Red) (Thermofisher) (1/50) and previously reported [59]. The percentage of immunostained tumor cells was scored as follows: 0, negative; <19%, weak: and >20%, positive. Representative images were acquired in a microscope (Olympus BX-61).

## Supplementary information

FigS1

FigS2

FigS3

FigS4

Table S1

Table S2

Table S3

Table S4

Table S5

Table S6

Figure legends Supp

Original Data File

checklist

## Data Availability

All data underlying the results are available as part of the article and no additional source data are required.
