# Supplementary figures and images for: VE-Cadherin modulates β-catenin/TCF-4 to enhance Vasculogenic Mimicry
Source: Cell Death Dis. 2023 Feb 17;14(2):135. doi: 10.1038/s41419-023-05666-7 (PMC9935922; doi:10.1038/s41419-023-05666-7)

Fig. S1

A

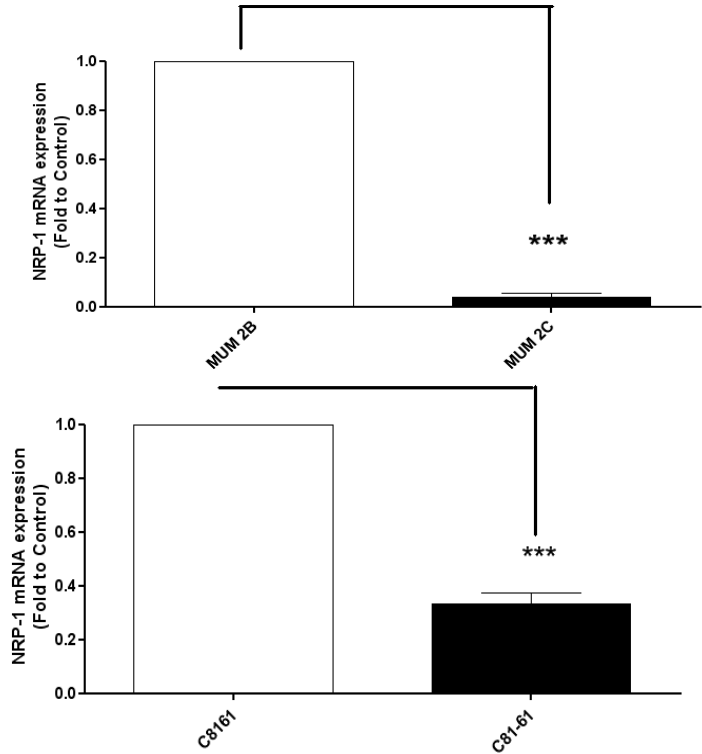

B

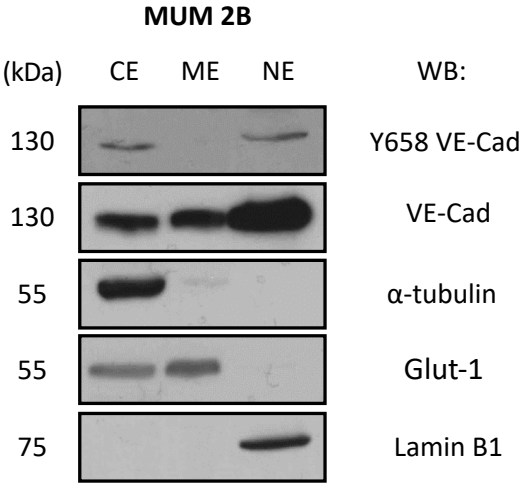

D

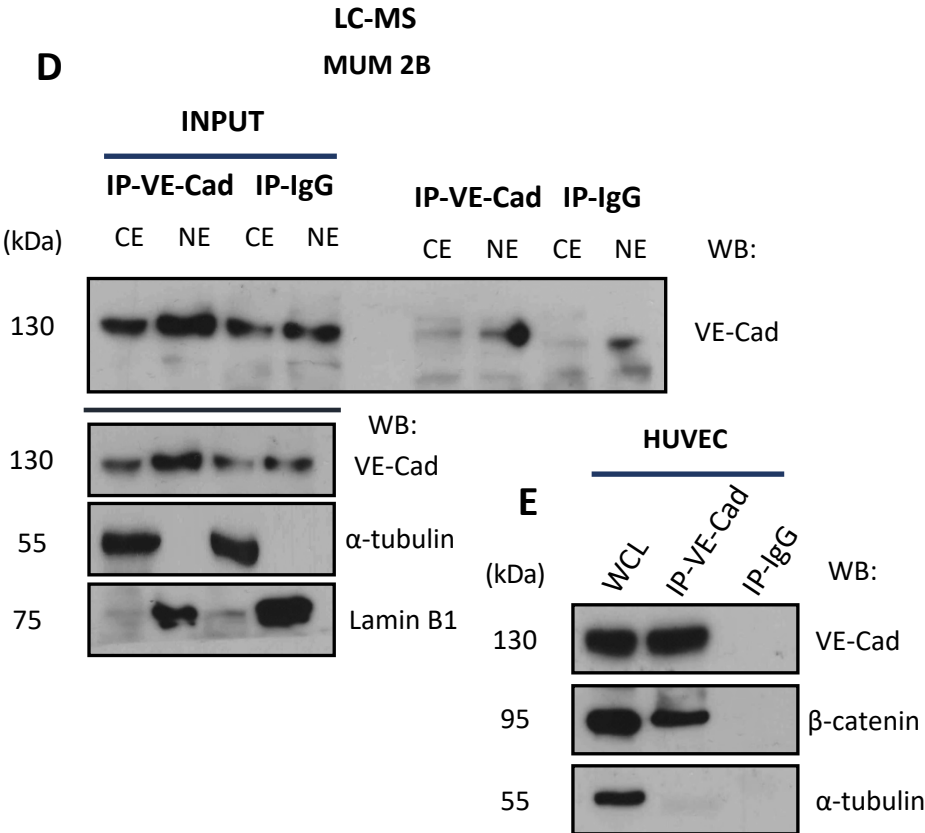

C

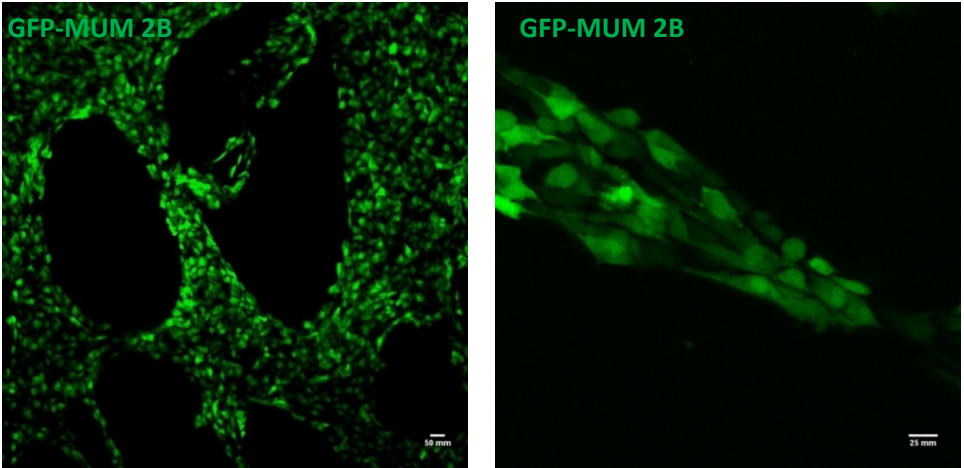

Supplement: Supplementary file 1 — FigS1 [file 41419_2023_5666_MOESM1_ESM.pdf]

**Fig. S2**

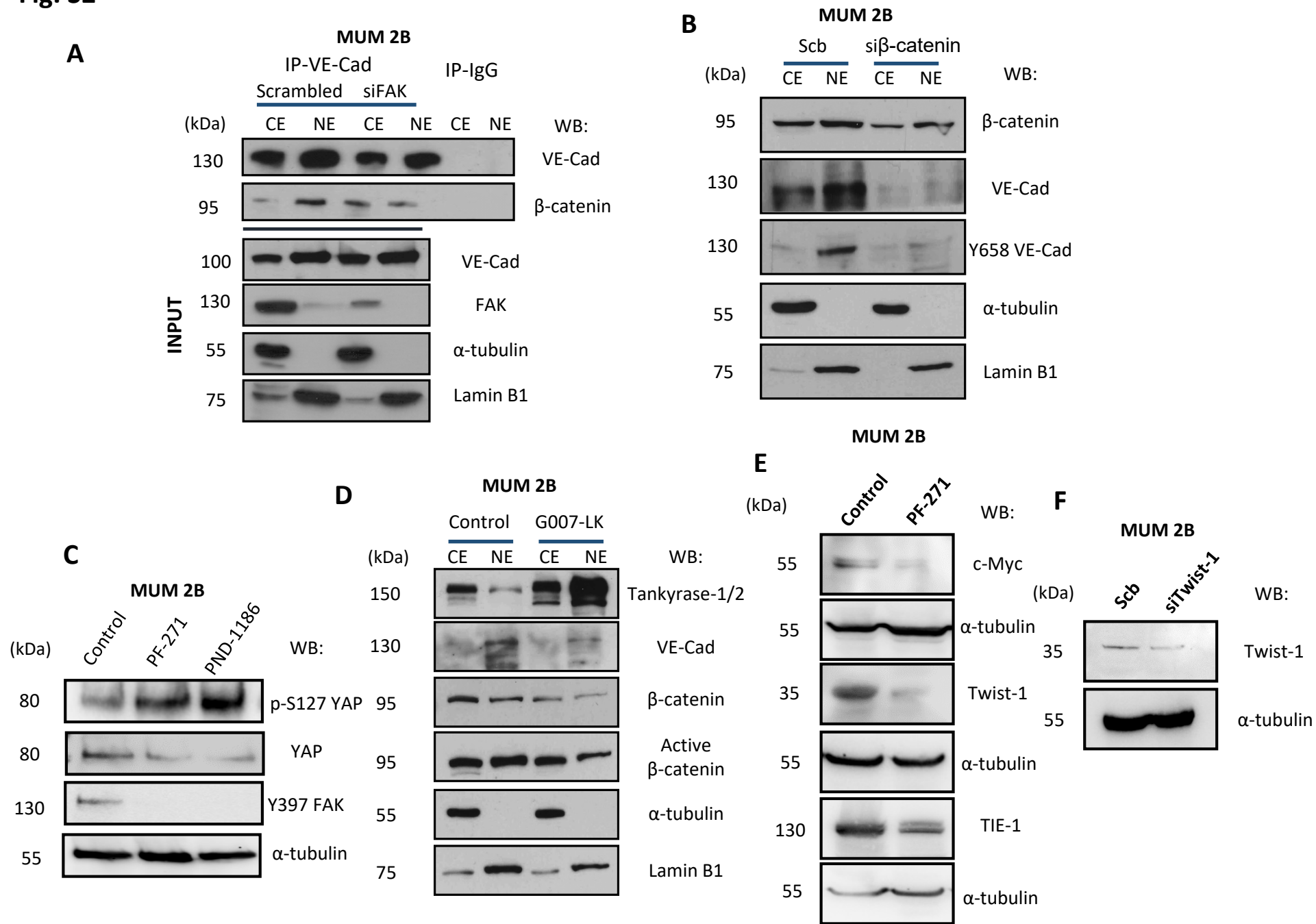

Supplement: Supplementary file 2 — FigS2 [file 41419_2023_5666_MOESM2_ESM.pdf]

Fig. S3

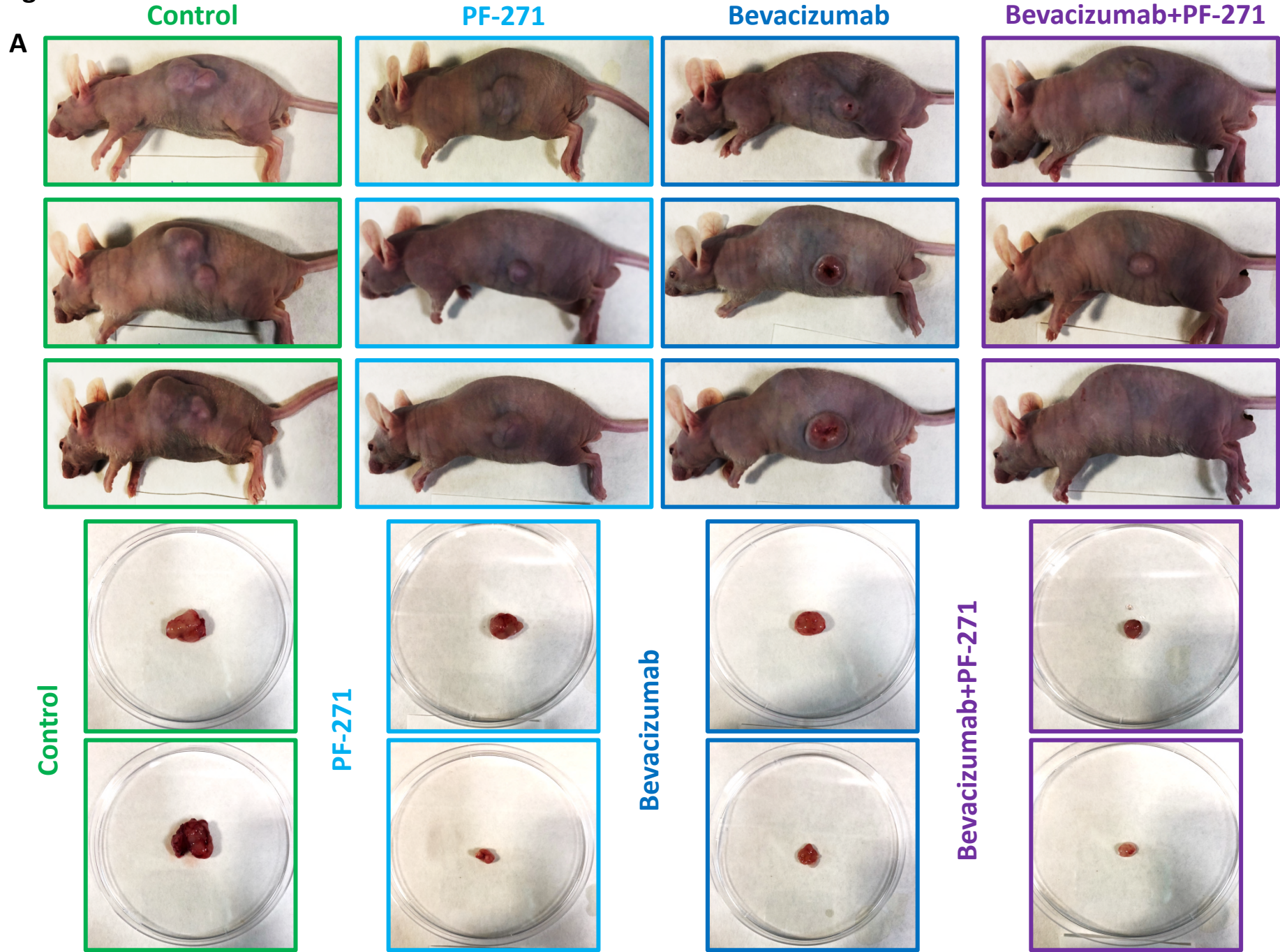

Supplement: Supplementary file 3 — FigS3 [file 41419_2023_5666_MOESM3_ESM.pdf]

Fig. S4

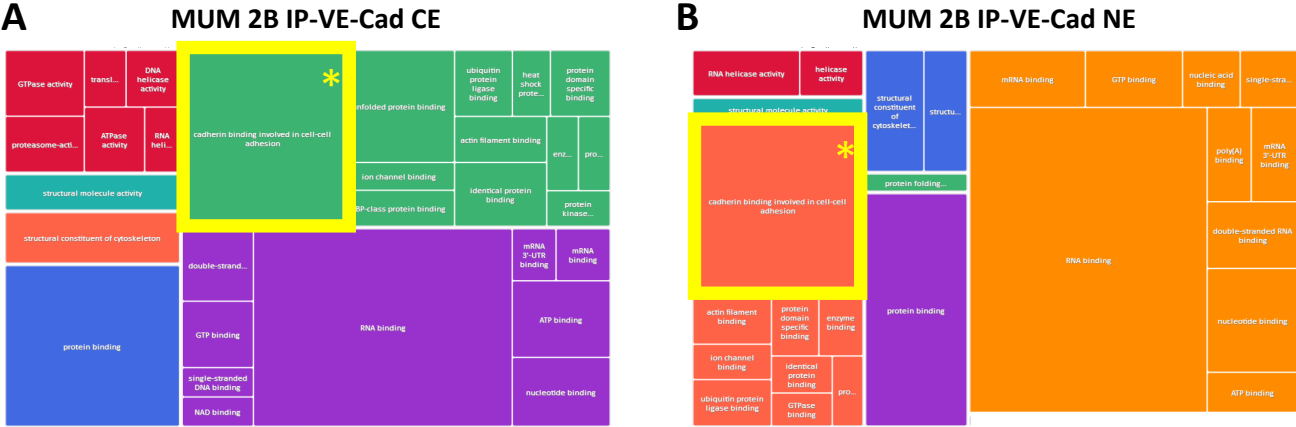

Cutaneous melanoma patients

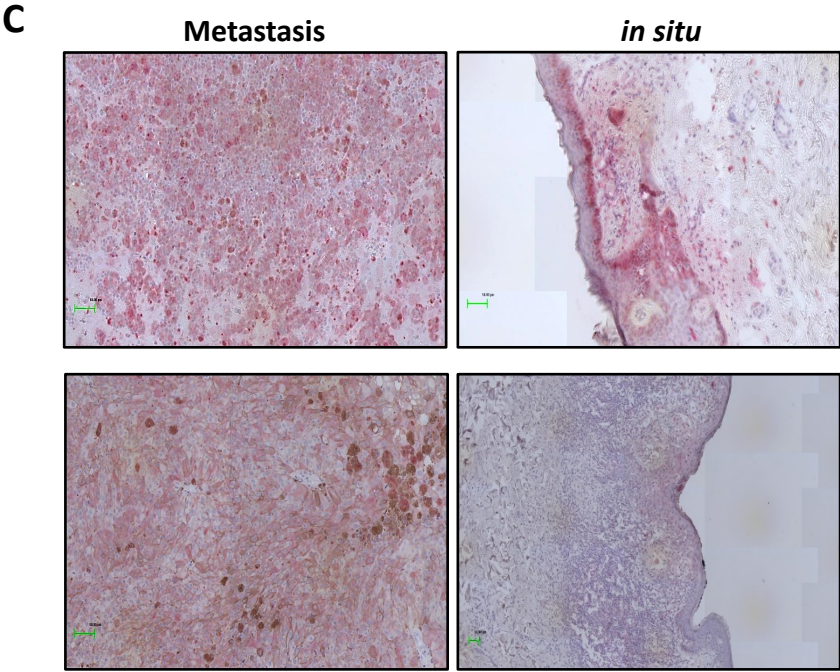

Supplement: Supplementary file 4 — FigS4 [file 41419_2023_5666_MOESM4_ESM.pdf]
