# Supplementary material for: VE-Cadherin modulates β-catenin/TCF-4 to enhance Vasculogenic Mimicry
Source: Cell Death Dis. 2023 Feb 17;14(2):135. doi: 10.1038/s41419-023-05666-7 (PMC9935922; doi:10.1038/s41419-023-05666-7)
Supplement: Supplementary file 11 — Figure legends Supp [file 41419_2023_5666_MOESM11_ESM.pdf]

Figure legends

Fig. S1: **Elevated expression of active β-catenin and Y397 phosphorylation of FAK in aggressive melanoma cells. A)** qPCR experiments showed strong downregulation of NRP-1 in MUM 2C vs. MUM 2B cells. Statistical analyses were conducted using Graph Pad Prism software. Statistical significance was calculated using a Student's *t* test (unpaired, two-tailed) with measurements from at least three independent experiments. **B)** cytosol-membrane-nucleus subfractionation in MUM 2B cells. **C)** GFP-MUM 2B cells were measured in an in vitro 3D angiogenesis assay to corroborate the VM formations. **D)** Immunoprecipitation of VE-Cadherin after cytosol-nucleus subractionation was corroborated by western blot to perform the LC-MS experiments in MUM 2B and **E)** a simple IP into HUVEC cells.

Fig. S2: **VE-Cadherin/β-catenin forms a complex in a FAK-dependent manner to enhance TCF-4 transcription activity. A)** Immunoprecipitation CE, NE of VE-cadherin after siFAK (50nM during 48h) in MUM 2B cells. **B)** subfractionation after siβ-catenin (50nM during 48h). **C)** FAKi with PF-271 and PND-1186 (1µM during 24h) treatment abolish YAP total expression by elevating the p-YAP S127, **D)** Western blot in subfraccionation experiments after G007-LK (5 µM during 24h) decrease VE-Cadherin and β-catenin protein expression. **E)** PF-271 (1µM during 24h) treatment reduced the expression of TIE-1, c-Myc, and Twist-1 in MUM 2B cells. **F)** scrambled and siTwist-1 (50nM during 48h) in MUM 2B cells.

Fig.S3: **FAK inhibition in combination prime line anti-angiogenesis Bevacizumab reduces the tumor growth in MUM 2B xenograft approach. A)** Representative pictures and animals of different tumors from treatment groups.

Fig.S4: **LC-MS experiments showed an elevated coupling of β-catenin/VE-cadherin complex in VM. A)** IP-VE-Cadherin CE Gene ontology/REVIGO representation graph or IP-VE-Cadherin NE **B),** default parameters were used to identify significantly enriched gene sets (FDR q< 0, 25). **C)** Immunochemistry analysis is performed to observe nuclear Y658 VE-Cadherin expression in melanoma in situ, metastasis patients. Consecutive sections are shown. Bars: 50μm
