# Supplementary material for: VE-Cadherin modulates β-catenin/TCF-4 to enhance Vasculogenic Mimicry
Source: Cell Death Dis. 2023 Feb 17;14(2):135. doi: 10.1038/s41419-023-05666-7 (PMC9935922; doi:10.1038/s41419-023-05666-7)

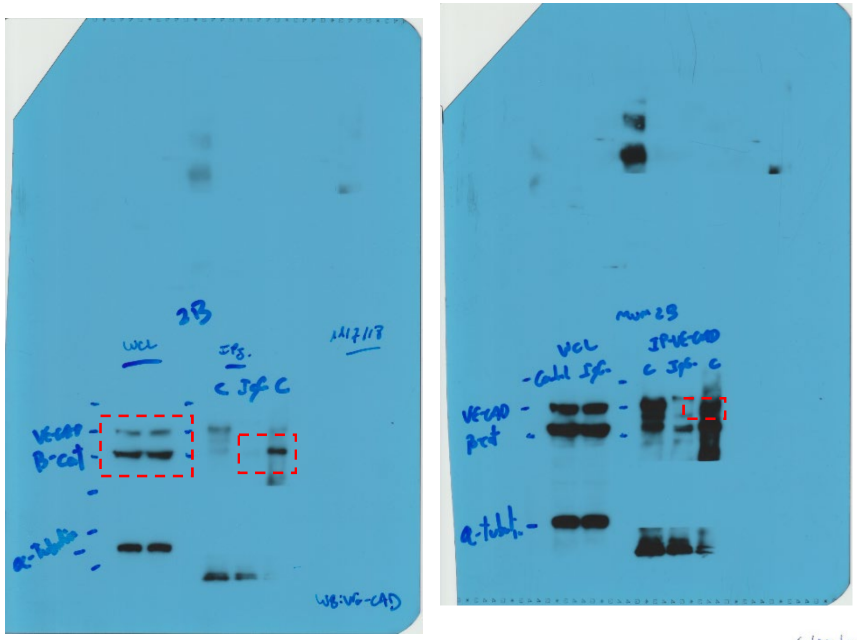

Fig.1A

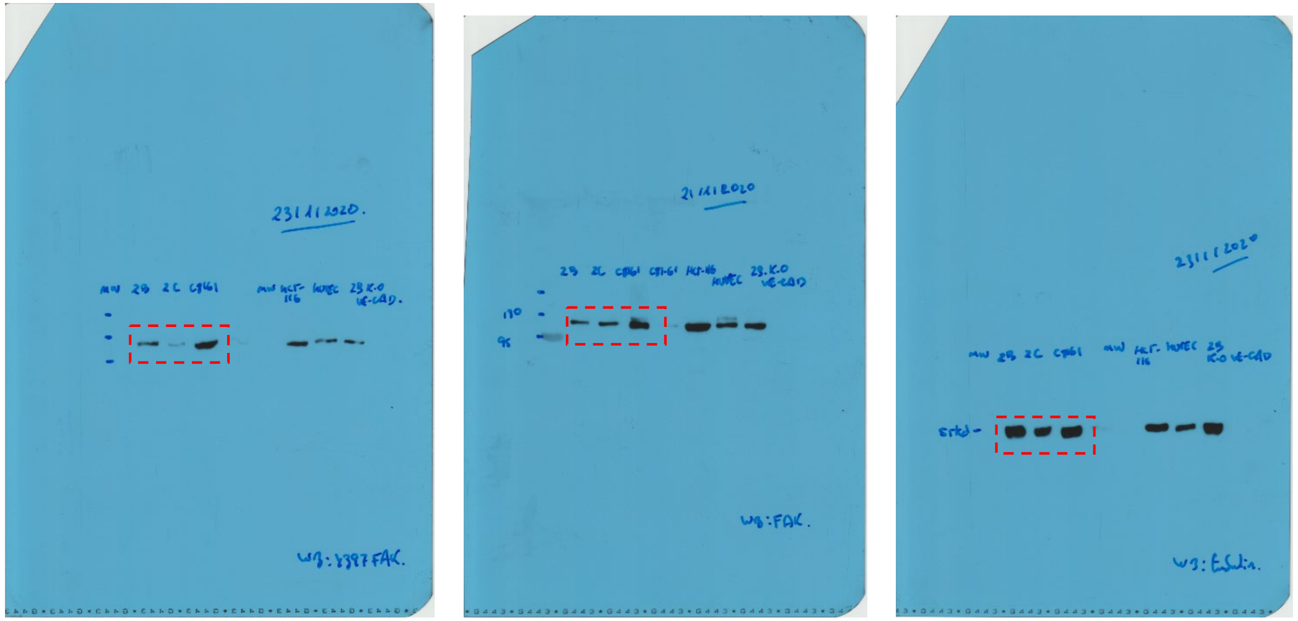

Fig.2A

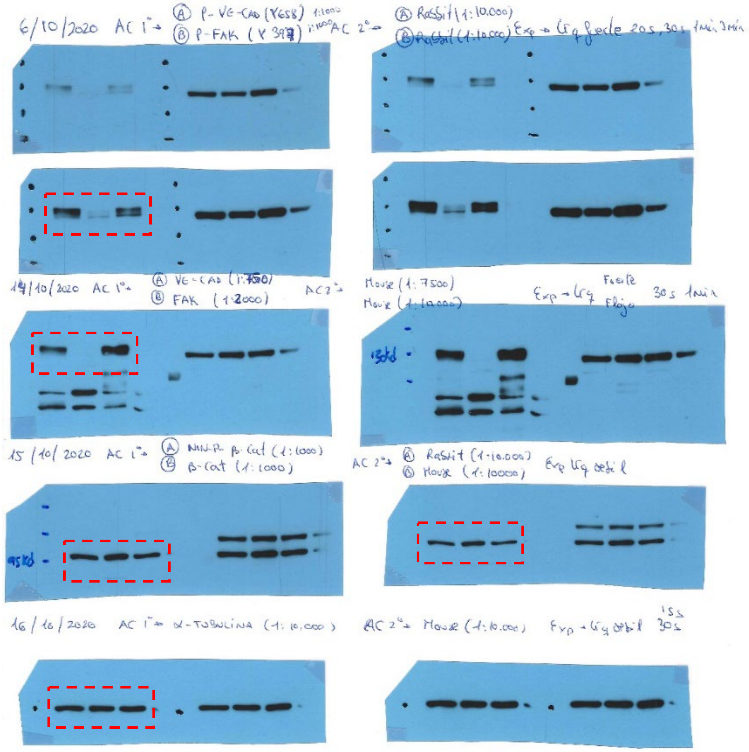

Fig.2A

Fig.2B

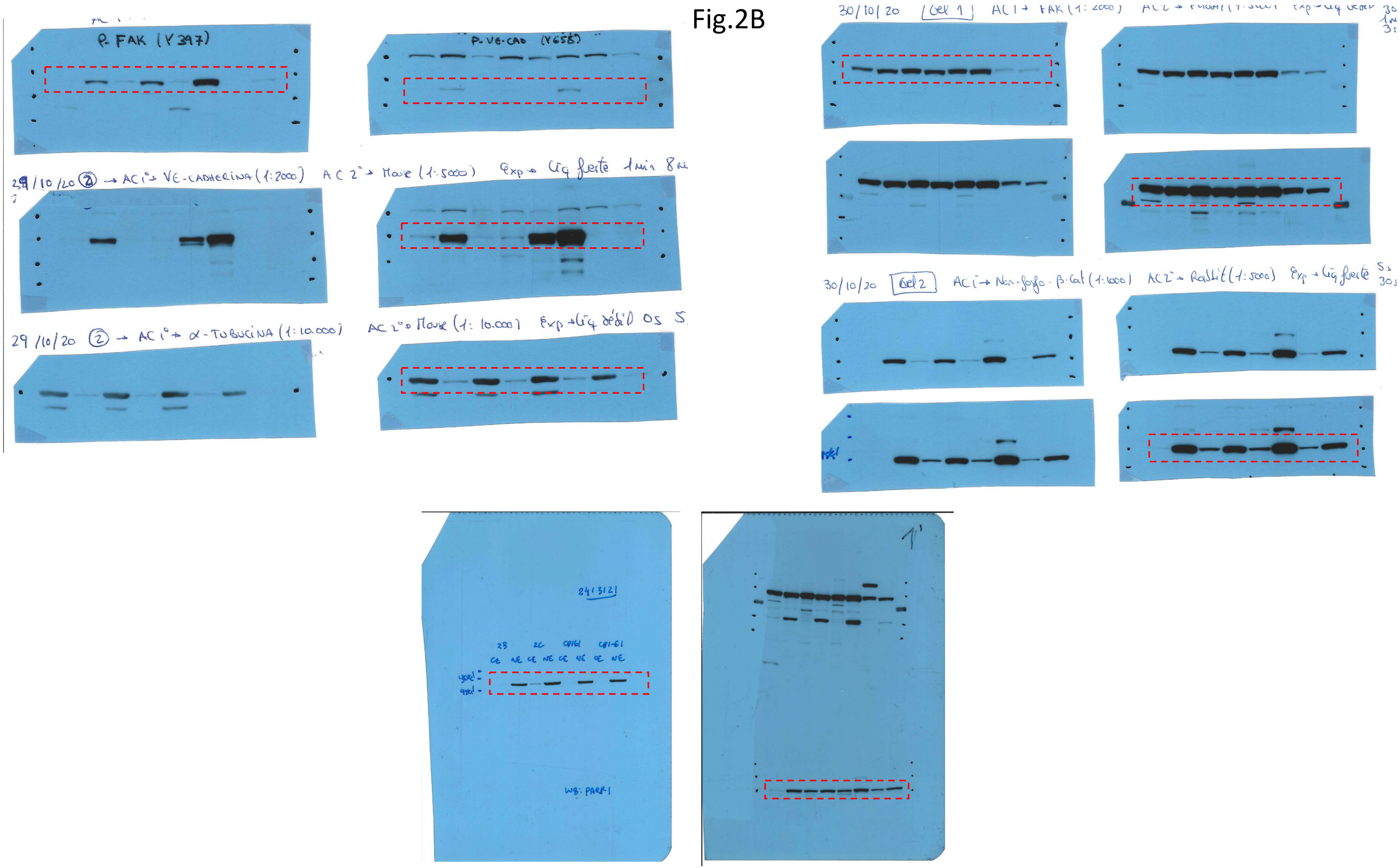

P-FAK (Y397)
P-VE-CAD (Y658)
29/10/20 ② → AC 1° → VE-CADHERINA (1:2000) AC 2° → Mouse (1:5000) Exp → Lig forte 1min
29/10/20 ② → AC 1° → α-TUBULINA (1:10.000) AC 2° → Mouse (1: 10.000) Exp → Lig débil 05 5
30/10/20 Gel 1 AC 1 → FAK (1: 2000)
30/10/20 Gel 2 AC 1 → Non-fosfo-β-Cat (1:1000) AC 2 → Rabbit (1:5000) Exp → Lig forte
24/3/21
2B 2C CB161 CB1-61
CE NE CE NE CE NE CE NE
WB: PARP1

Fig.3A,B

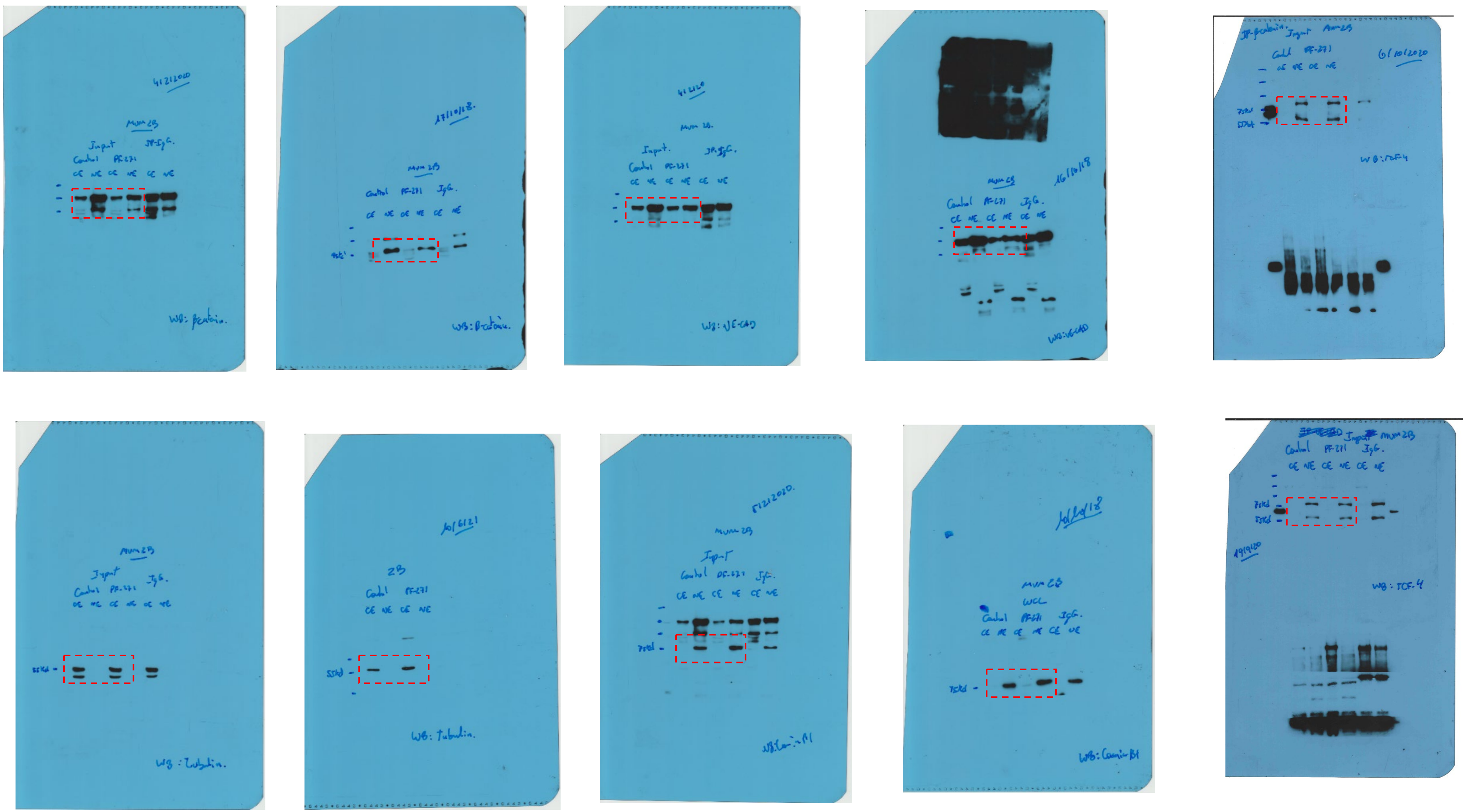
MVM 2B
Input
Control
PF-271
IgG.
CE NE CE NE CE NE
WB: Tubulin.
WB: Tubulin.
WB: TCF-4
55kd
75kd

Fig.3A,B

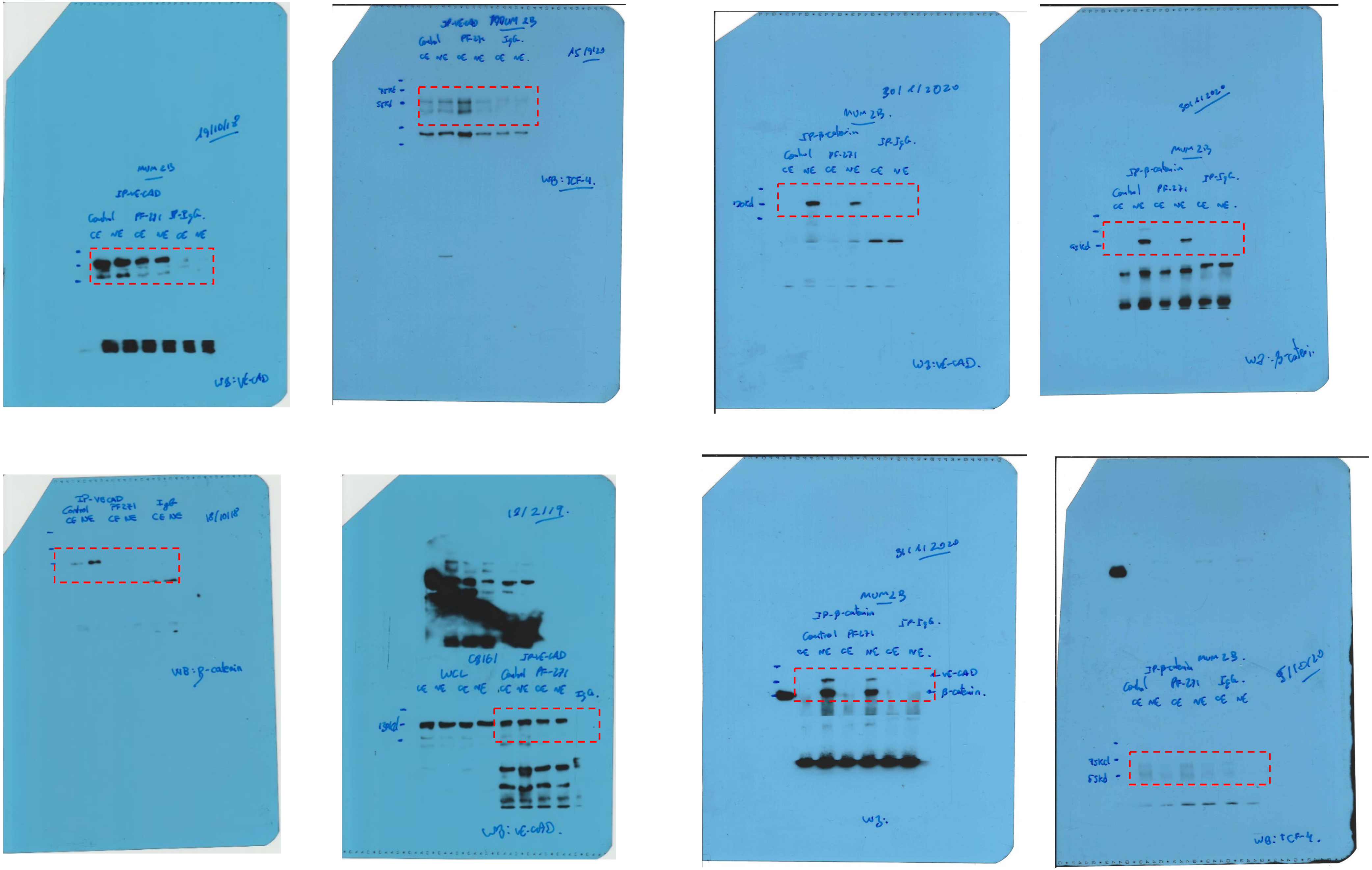

19/10/18
MUM 2B
IP-VE-CAD
Control PF-271 IP-IgG.
CE NE CE NE CE NE
WB: VE-CAD
15/1/20
Control PF-271 IgG.
CE NE CE NE CE NE.
WB: TCF-4.
30/1/2020
MUM 2B.
IP-β-catenin
IP-IgG.
Control PF-271
CE NE CE NE CE NE
WB: VE-CAD.
WB: β-catenin.
18/10/18
IP-VE CAD
Control PF271 IgG
CE NE CE NE CE NE
WB: β-catenin
12/2/19.
WCL
CE NE CE NE
IP-VE-CAD
Control PF-271
CE NE CE NE
IgG.
WB: VE-CAD.
MUM 2B
IP-β-catenin
Control PF-271
IP-IgG.
CE NE CE NE CE NE.
VE-CAD
β-catenin.
WB:
IP-β-catenin
MUM 2B.
Control PF-271 IgG.
CE NE CE NE CE NE
WB: TCF-4.

Fig.3C,D

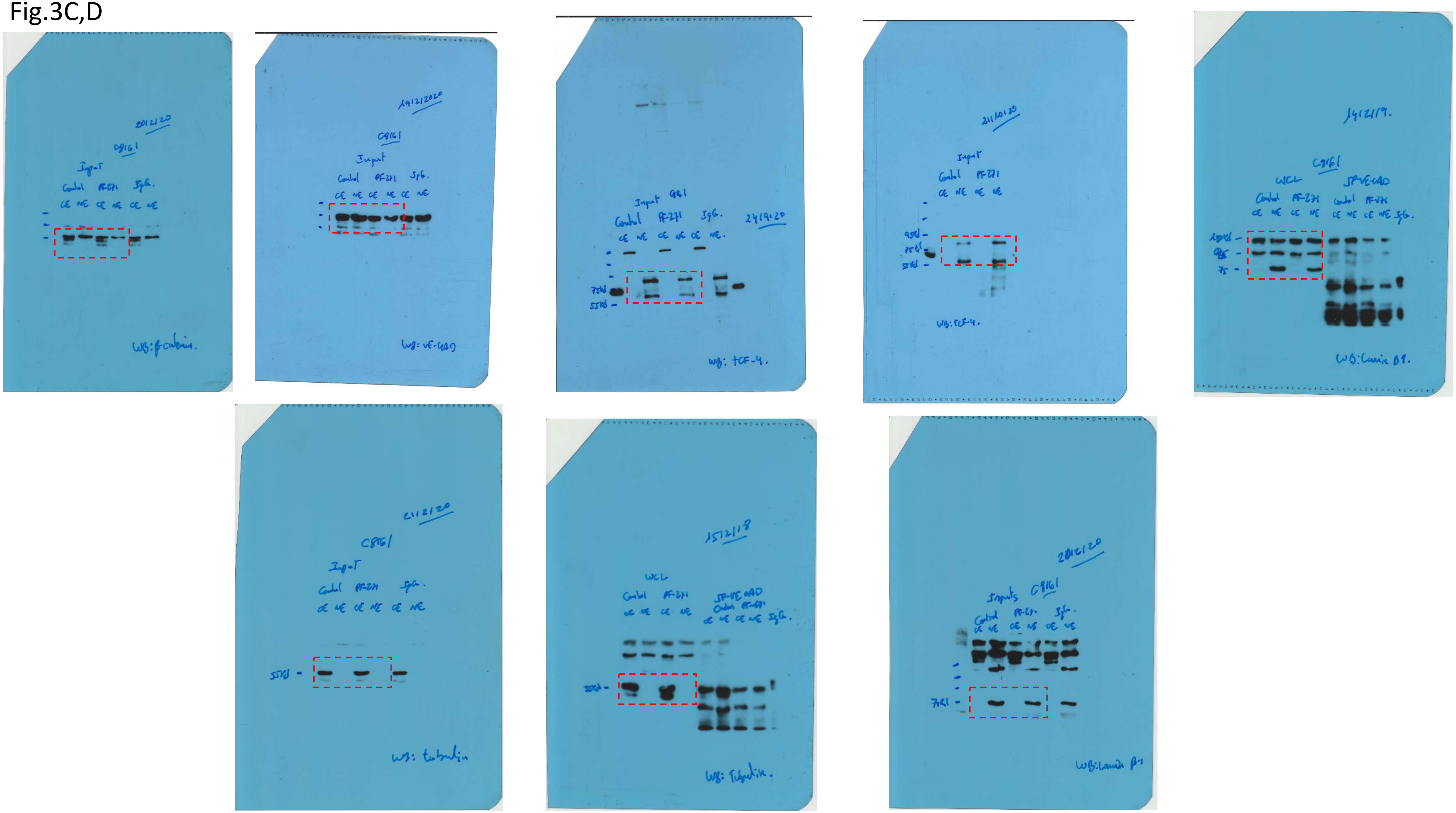

Fig.3C,D

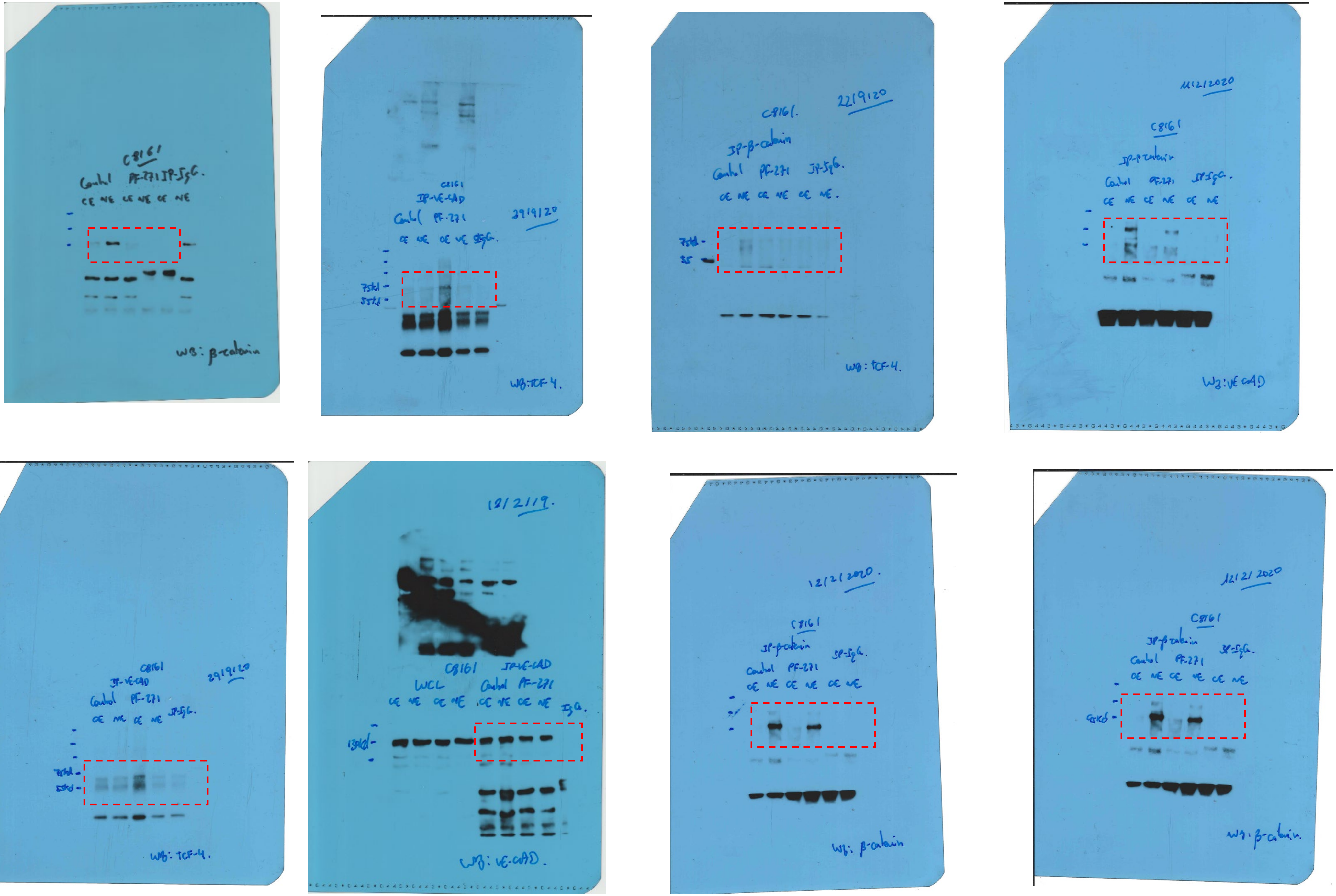
C8161
WB: β-catenin
WB: TCF-4
WB: TCF-4
WB: VE CAD
WB: TCF-4
WB: VE-CAD
WB: β-catenin
WB: β-catenin

Fig.5C

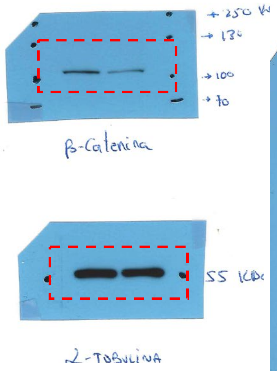

Fig.6A

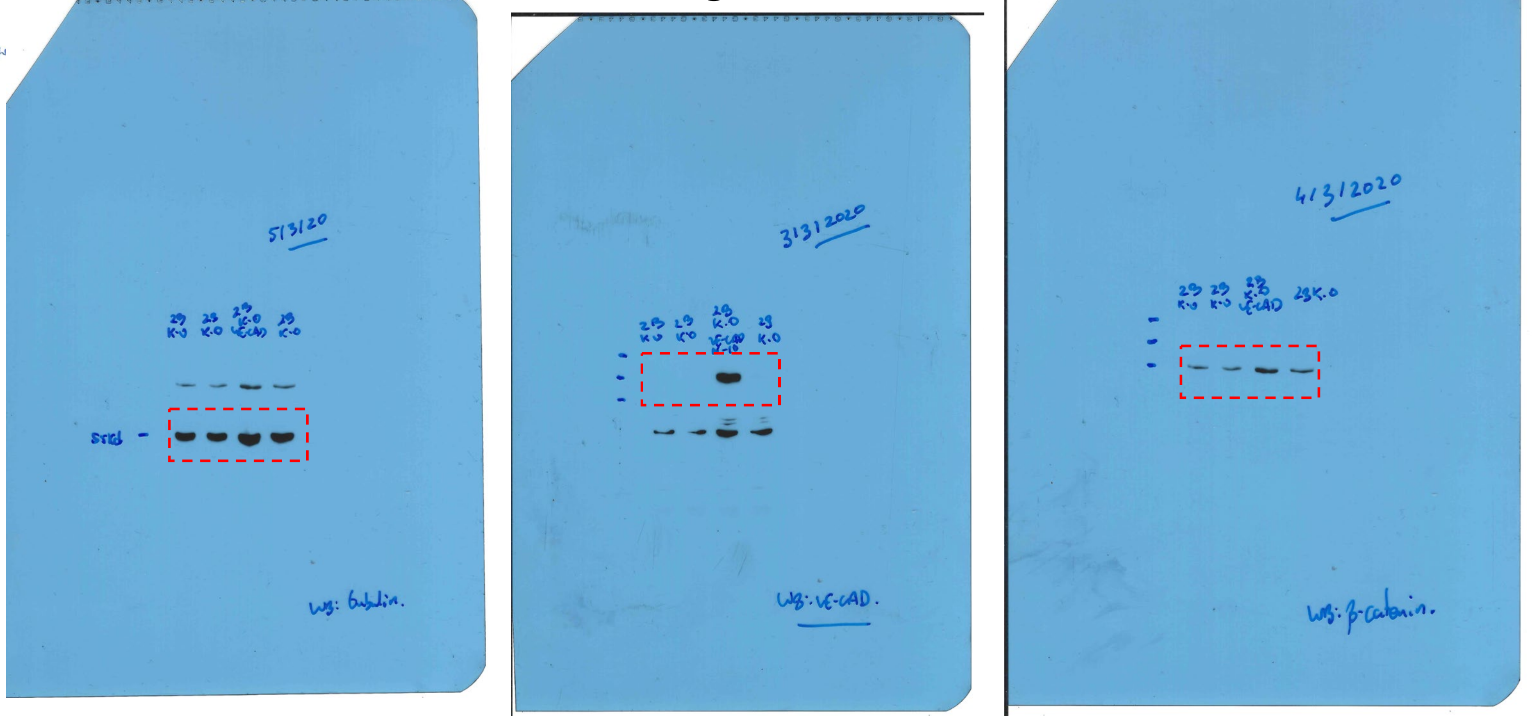

Fig.6B

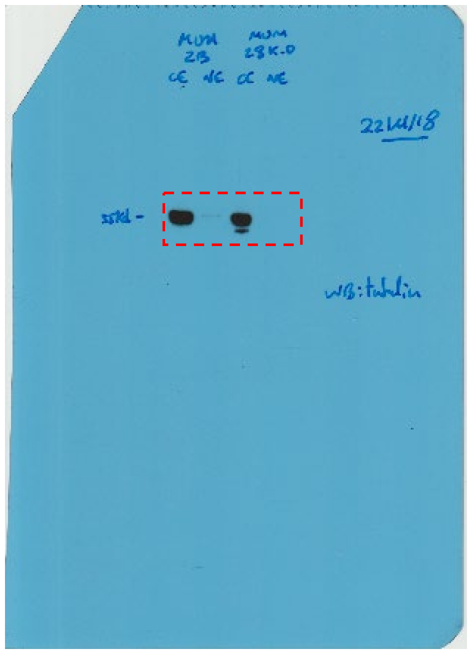

Fig.6B-C

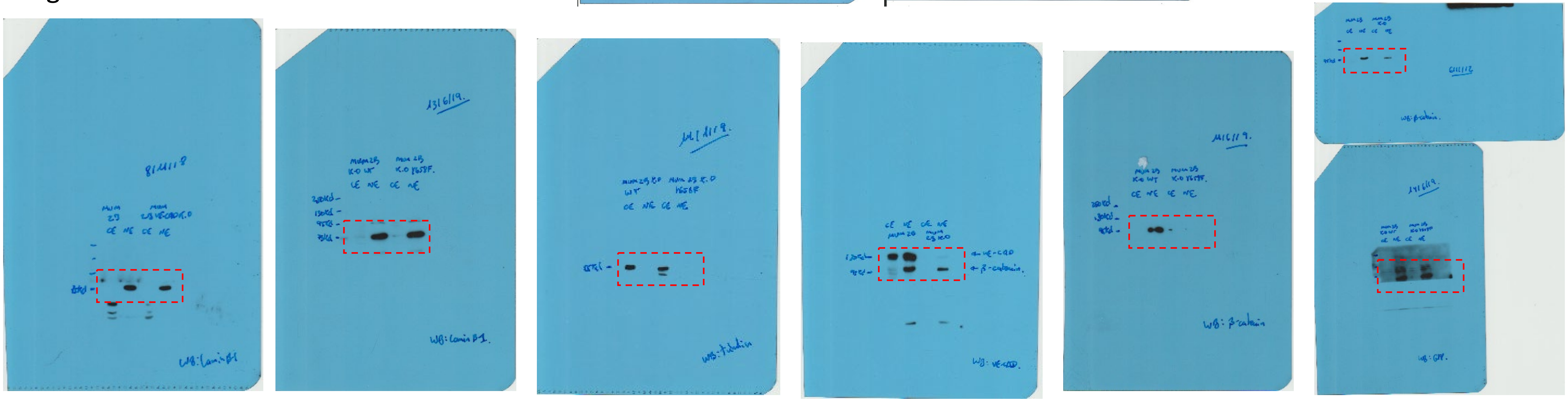

Fig.6D

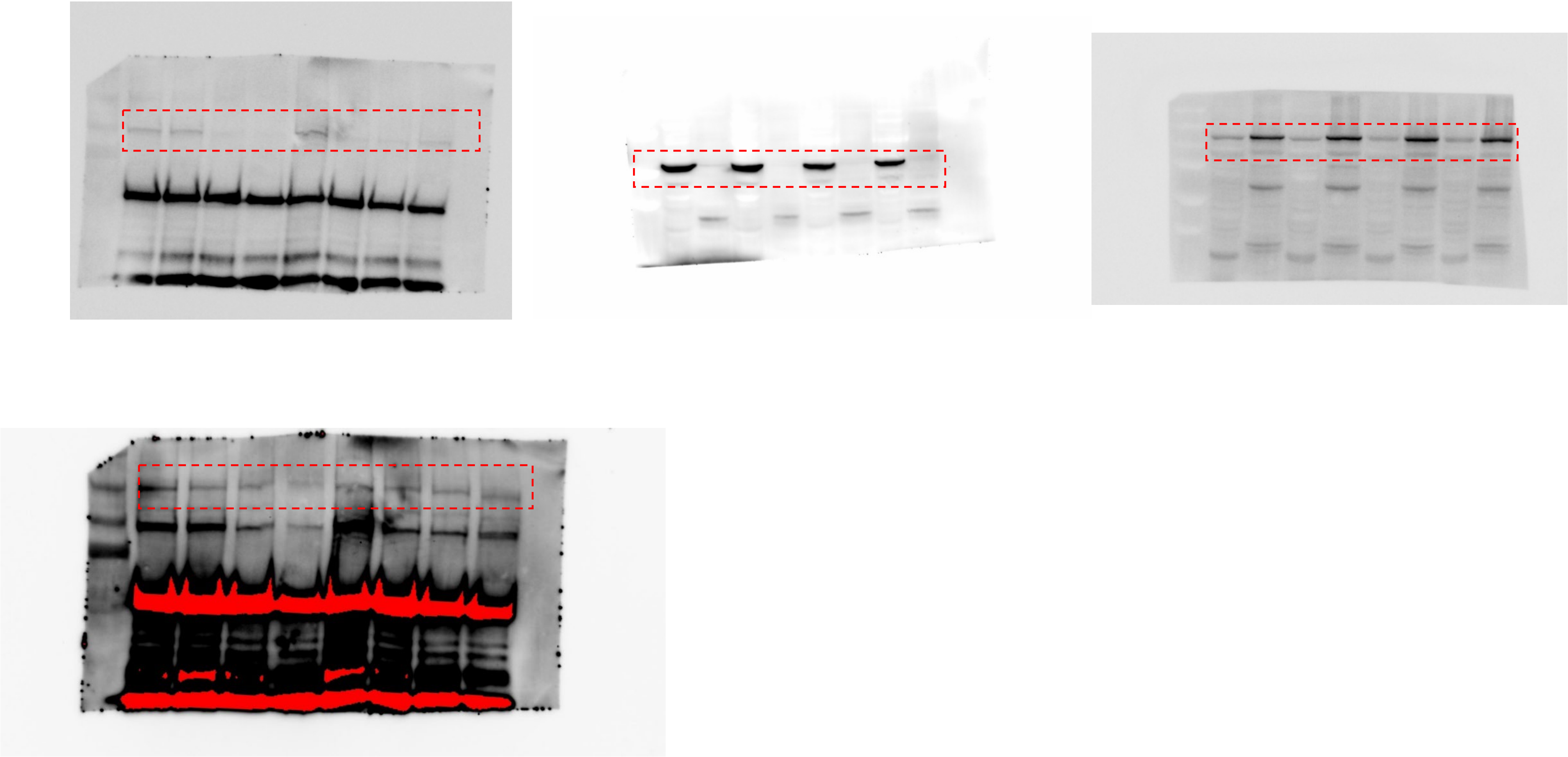

Fig.S1

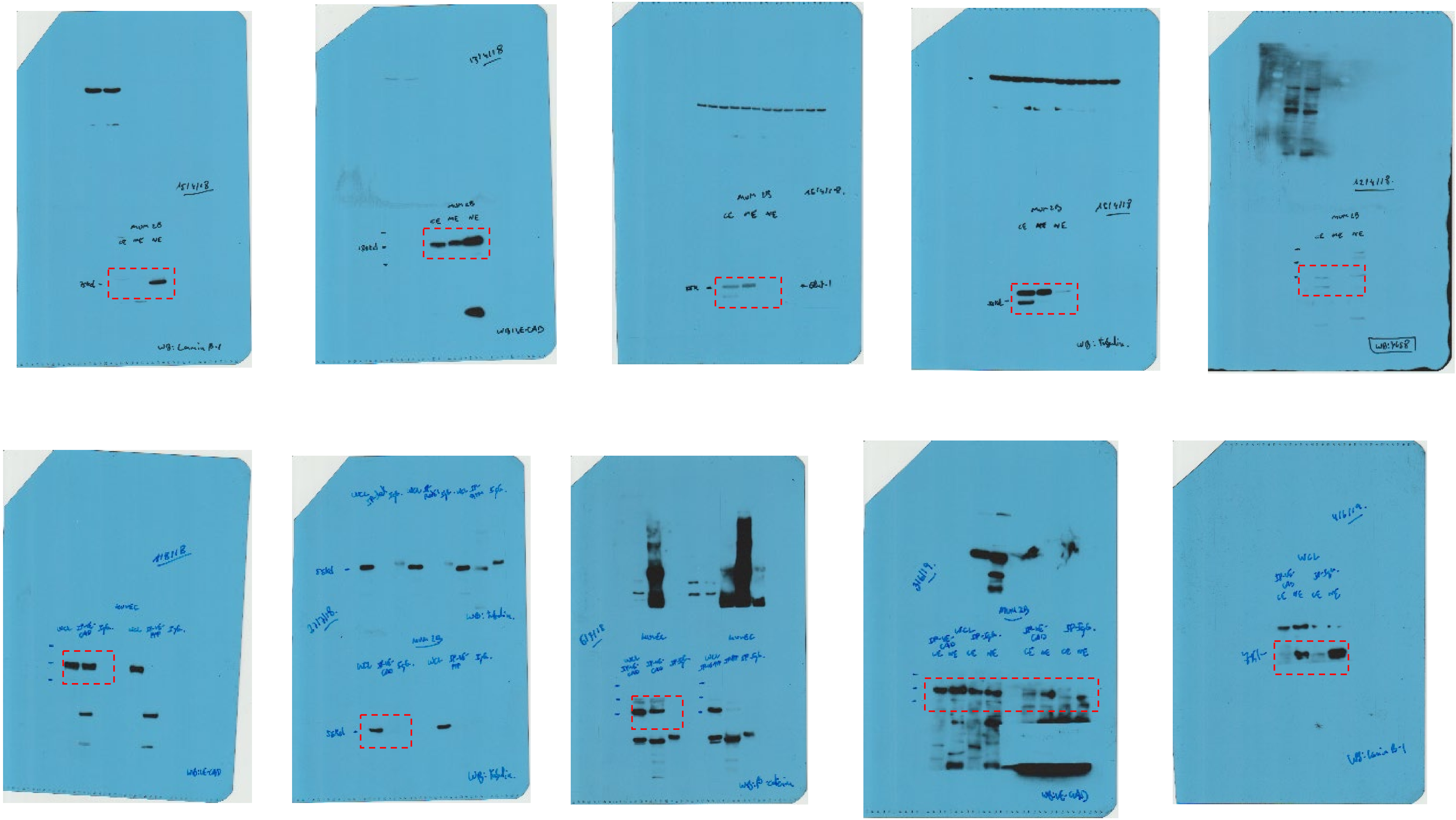

Fig.S2

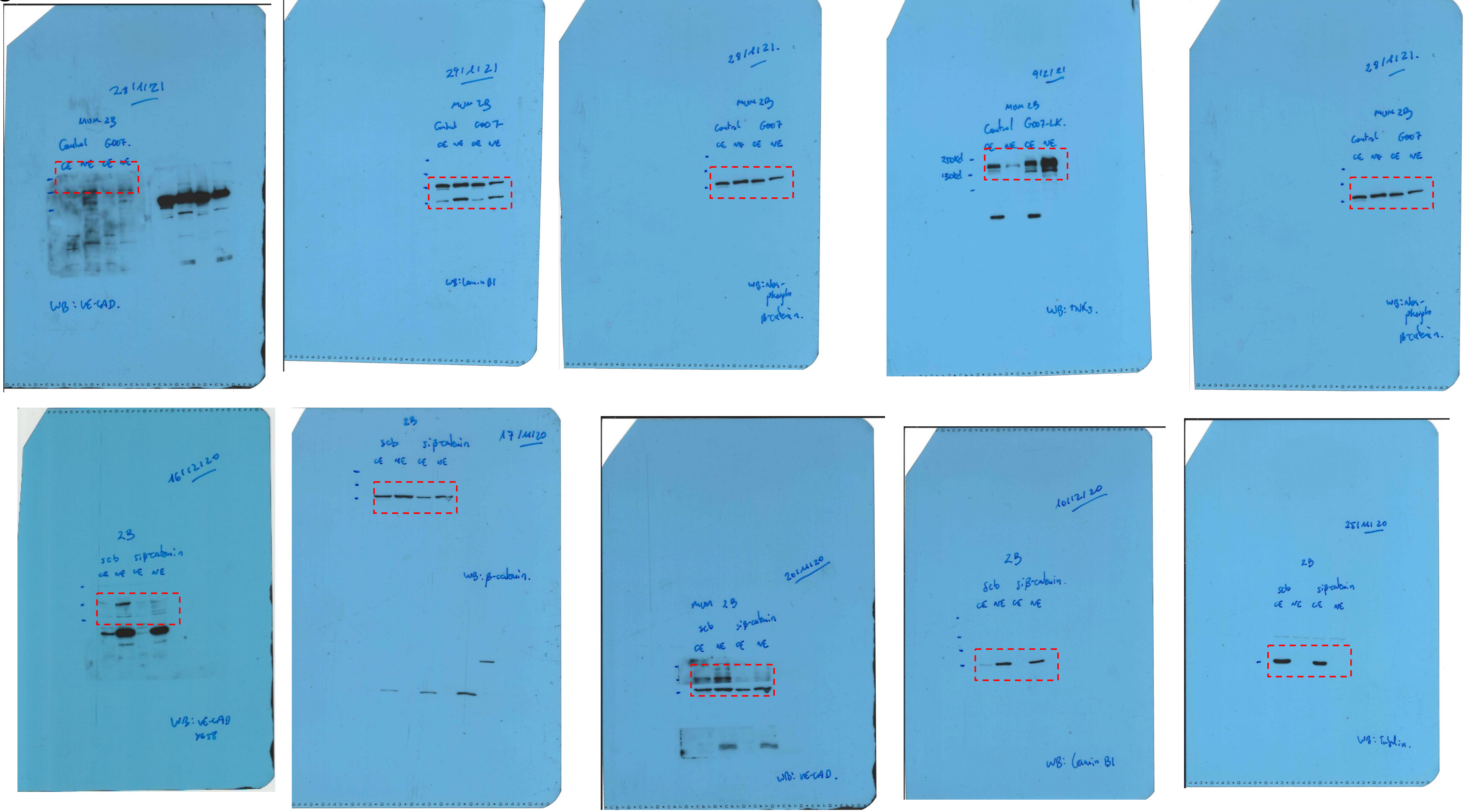
MUM 2B
Control
G007
CE NE CE NE
WB: VE-CAD.
WB: Lamin B1
WB: TNKS.
250kd
130kd
2B
scb
si β-catenin
WB: β-catenin.
WB: VE-CAD
Y658
WB: Lamin B1
WB: Tubulin.

Fig.S2

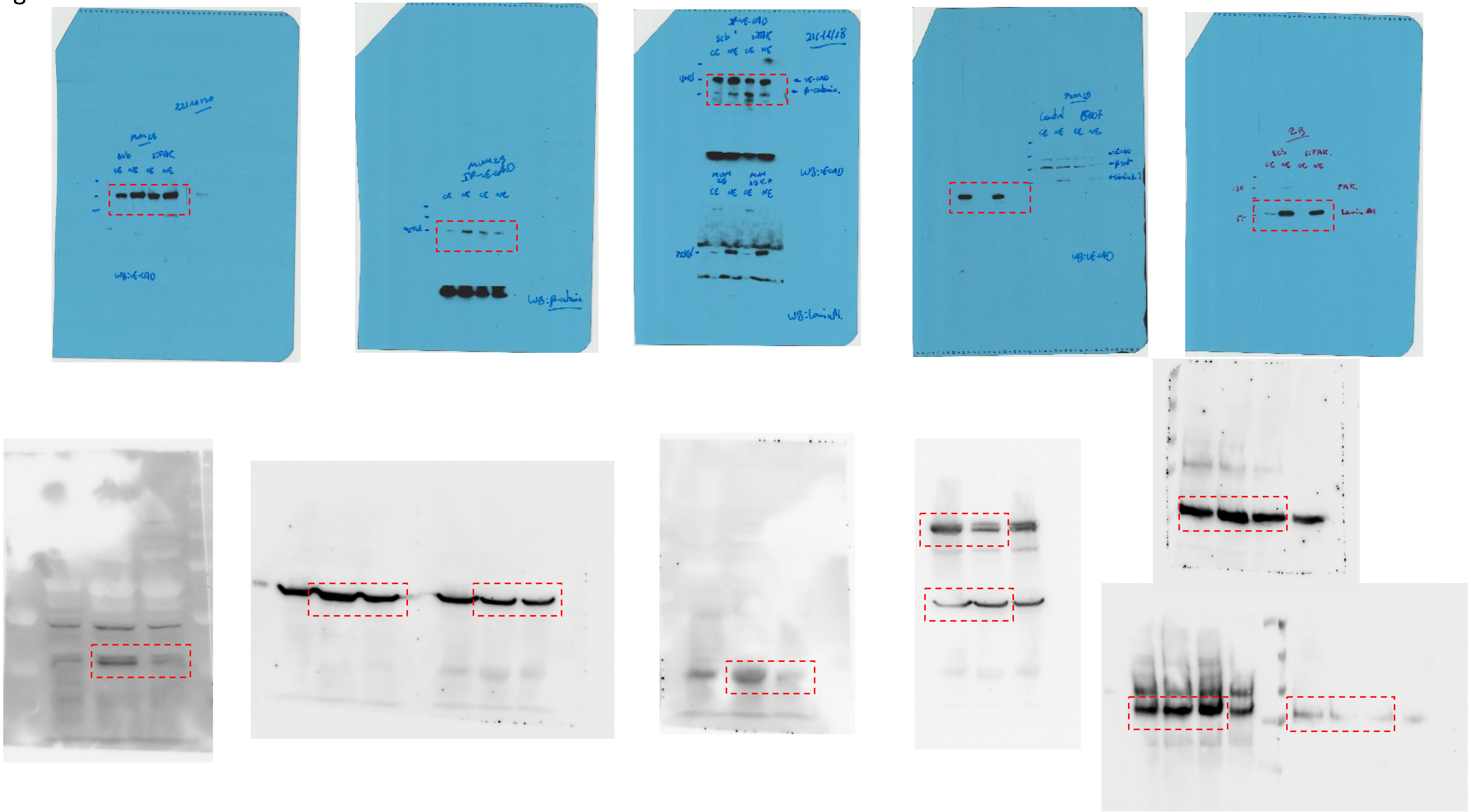

Supplement: Supplementary file 12 — Original Data File [file 41419_2023_5666_MOESM12_ESM.pdf]
